# Nephroprotective Effect of *Pleurotus ostreatus* and *Agaricus bisporus* Extracts and Carvedilol on Ethylene Glycol-Induced Urolithiasis: Roles of NF-κB, p53, Bcl-2, Bax and Bak

**DOI:** 10.3390/biom10091317

**Published:** 2020-09-14

**Authors:** Osama M. Ahmed, Hossam Ebaid, El-Shaymaa El-Nahass, Mahmoud Ragab, Ibrahim M. Alhazza

**Affiliations:** 1 Physiology Division, Zoology Department, Faculty of Science, Beni-Suef University, Beni-Suef P.O. 62521, Egypt; 2 Department of Zoology, College of Science, King Saud University, P.O. Box 2455, Riyadh 11451, Saudi Arabia; ihazza@ksu.edu.sa; 3 Department of Zoology, Faculty of Science, El-Minia University, Minya P.O. 61519, Egypt; 4 Department of Pathology, Faculty of Veterinary Medicine, Beni-Suef University, Beni-Suef P.O. 62521, Egypt; shima_k81@vet.bsu.edu.eg; 5 Sohag General Hospital, Sohag 42511, Egypt; mrjb4225@gmail.com; 6 The Scientific Office of Pharma Net Egypt Pharmaceutical Company, Nasr City, Cairo 11371, Egypt

**Keywords:** *Pleurotus ostreatus*, *Agaricus bisporus*, carvedilol, hyperoxaluria, urolithiasis, ethylene glycol

## Abstract

This study was designed to assess the nephroprotective effects of *Pleurotus ostreatus* and *Agaricus bisporus* aqueous extracts and carvedilol on hyperoxaluria-induced urolithiasis and to scrutinize the possible roles of NF-κB, p53, Bcl-2, Bax and Bak. Phytochemical screening and GC-MS analysis of mushrooms’ aqueous extracts were also performed and revealed the presence of multiple antioxidant and anti-inflammatory components. Hyperoxaluria was induced in Wistar rats through the addition of 0.75% (*v*/*v*) ethylene glycol in drinking water for nine weeks. The ethylene glycol-administered rats were orally treated with *Pleurotus ostreatus* and *Agaricus bisporus* aqueous extracts (100 mg/kg) and carvedilol (30 mg/kg) daily during the last seven weeks. The study showed that *Pleurotus ostreatus*, *Agaricus bisporus* and carvedilol all successfully inhibited ethylene glycol-induced histological perturbations and the elevation of serum creatinine, serum urea, serum and urinary uric acid, serum, urinary and kidney oxalate, urine specific gravity, kidney calcium, kidney NF-κB, NF-κB p65, NF-κB p50, p53, Bax and Bak expressions as well as serum TNF-α and IL-1β levels. Moreover, the treatment decreased the reduction in urinary creatinine, urinary urea, ratios of urinary creatinine to serum creatinine and urinary urea to serum urea, Fex Urea and Bcl-2 expression in kidney. In conclusion, although *Pleurotus ostreatus* and *Agaricus bisporus* extracts and carvedilol all significantly inhibited the progression of nephrolithiasis and showed nephroprotective effects against ethylene glycol-induced kidney dysfunction, *Pleurotus ostreatus* and *Agaricus bisporus* seemed to be more effective than carvedilol. Moreover, the nephroprotective effects may be mediated via affecting NF-κB activation, extrinsic apoptosis and intrinsic apoptosis pathways.

## 1. Introduction

Urolithiasis is a urinary stone disease involving the calcifications in the kidney [1]. In addition, urolithiasis is a very painful and the third common disorder of urinary system [2,3]. Epidemiological studies have revealed that the majority of stones are commonly composed of calcium oxalate (CaOx) [4,5]. The risk of developing urolithiasis appears to be associated with a multitude of genetic, physiological and nutritional disorders, ranging from general hyperoxaluria to obesity [6].

Hyperoxaluria describes the occurrence of high oxalate concentrations in urine. Although hyperoxaluria is most often simply a result of excess dietary oxalate intake, with research suggesting that dietary oxalate accounts for as much as 50% of the total urinary oxalate, there is also a genetic form of the condition, known as primary hyperoxaluria [7,8,9,10,11]. Hyperoxaluria is, in turn, an important risk factor for urolithiasis and more than 80% of the uroliths consist of CaOx, either alone or mixed with calcium phosphate [12,13,14].

The exposure of the renal epithelial cells to high levels of oxalate and crystals of CaOx/calcium phosphate generates excess reactive oxygen species (ROS), causing injury and inflammation [15]. Epithelial cell injury facilitates the events of CaOx crystal nucleation and aggregation and promotes crystal retention in renal tubules, which is crucial for subsequent stone development [5,16,17,18]. In humans, the accumulation of oxalate causes a number of pathological conditions, which include hyperoxaluria, the formation of CaOx kidney stones (urolithiasis) and renal failure [19]. The regulation of the genes that encode proteins involved in immune and inflammatory responses (i.e., cytokines, chemokines, growth factor immune receptors, cellular ligands and adhesion molecules) is controlled by nuclear factor-kappa B (NF-κB) [20]. Thus, NF-κB plays a pivotal role in control of the inflammation in hyperoxaluria and obstructive nephropathy [21]. On the other hand, it was speculated that hyperoxaluria induced apoptosis in renal tubular epithelial cells as an end stage of crystal–cell interaction [22]. The target genes involved in apoptosis are induced after activation of p53 upon DNA damage [23]. Bcl-2, which is an integral mitochondrial membrane protein, blocks apoptosis induced by a wide array of death signals [24]. 

Although different treatment strategies for urolithiasis have evolved over the years, discrepancies exist concerning the clinical indications and the efficacy of each of these treatment options [25]. Current medical approaches for therapies of urolithiasis are based on carefully constructed and rational modifications of urinary biochemistry as well as physical chemistry to minimize stone secondary risks rather than etiologies. Thus, the search for product of natural sources that prevent the causes and progress or even the recurrence of nephrolithiasis has attracted many researchers [26,27,28,29].

Mushrooms have long had a role as a branch of alternative medicine [30] and as functional diets [31]. In particular, *Pleurotus ostreatus* (*P. ostreatus*) has been confirmed to have medicinal effects, spanning anti-carcinogenic, anti-hypercholesterolemic, anti-inflammatory, anti-oxidative, immuno-stimulating and anti-viral properties and ability to regulate blood lipid and glucose levels [32,33,34,35,36]. Another important medicinal mushroom is *Agaricus bisporus* (*A. bisporus*), which has been demonstrated to possess various valuable biological properties including anti-aromatase, anti-inflammatory, antitumor, antimicrobial, antioxidant and immunomodulatory activities [37,38,39,40].

Carvedilol, meanwhile, is a multifunctional neurohormonal antagonist that has been shown to provide greater benefit than classical β-blockers because of its antioxidant effects, which work in synergy with its nonspecific α1- and β-blocking actions [41]. It has also been reported that carvedilol has anti-inflammatory activities in addition to its antioxidant properties [42]. Carvedilol is of interest in this study since it has been shown to protect against nephrotoxicity induced by ferric nitrilotriacetate in rats [43] as well as to improve renal function by ameliorating renal ischemia-reperfusion injury [44]. Finally, it has been speculated that carvedilol is a very promising candidate for clinical trials in future as a nephroprotector in patients treated with the nephrotoxic drug, cisplatin [45].

In conductance with the previous literature, the present study aimed to assess the protective effects of *P. ostreatus* and *A. bisporus* aqueous extracts and carvedilol on hyperoxaluria-induced nephrolithiasis in Wistar rats and to elucidate the probable implications of NF-κB, NF-κB p65, NF-κB p50, p53, Bcl-2, Bax and Bak in their mechanisms of action. 

## 2. Materials and Methods

### 2.1. Animals and Housing

Male Wistar rats of weight ranging from 115 to 140 g were obtained from the National Research Center (NRC), El-Tahrir Street, Dokki, Egypt. To exclude any intercurrent infections, rats were kept overseen for ten days before starting the experiment. The rats were housed in cages with good aerated covers at room temperature (20–25 °C) and 12-h light/dark cycle, supplied with excess food (balanced standard diet) and water ad libitum. All animal procedures were approved by Experimental Animal Ethics Committee for use and care of animals, Faculty of Science, Beni-Suef University, Egypt (Ethical Approval number: BSU/FS/2015/19). All efforts were done to minimize the pain, discomfort and suffering of animals.

### 2.2. Mushrooms

**Figure 1:**
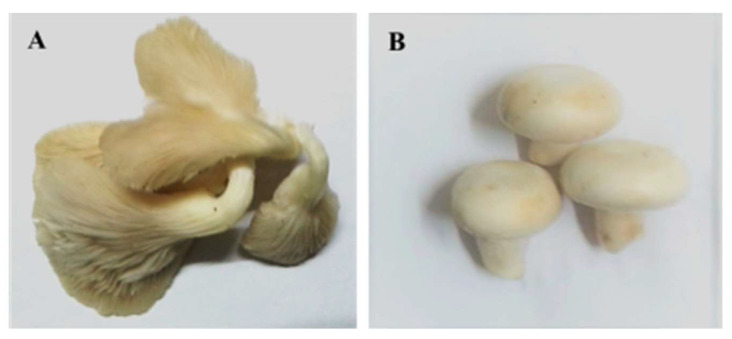
*P. ostreatus* (**A**); and *A. bisporus* (**B**).

Oyster mushroom (*P. ostreatus*) and button mushroom (*A. bisporus*) (Figure 1 and Figure S1) were supplied from Agriculture Research Center, Giza, Egypt. The two mushrooms were identified by Dr. Fathy Ragab, Food Technology Research Institute, Agriculture Research Center, Giza, Egypt. Fruiting bodies were cut into small pieces and dried at shaded good aerated area.

### 2.3. Preliminary Phytochemical Screening of Mushrooms

The tests, performed according to Claus [46], were used to detect different active principles including glycosides, carbohydrates, alkaloids, flavonoids, saponins, tannins, resins, unsaturated sterols and triterpenes.

### 2.4. Preparation of Mushroom Aqueous Extracts

The dried fruiting bodies of *P. ostreatus* and *A. bisporus* were roughly cut and ground with an electric grinder. *P. ostreatus* and *A. bisporus* aqueous extracts were prepared by adding certain weight of the powdered mushroom to certain volume of boiling distilled water (2% *w*/*v*) and was soaked for 15 min then filtrated. The resulting filtrates of *P. ostreatus* and *A. bisporus* were orally given to the rats at dose level of 100 mg/kilogram body weight (kg b.w.) daily by oral gavage for seven weeks. The doses of *P. ostreatus* and *A. bisporus* were orally administered, in the present study, according to the doses used by previous publications [47,48].

### 2.5. Gas Chromatography–Mass Spectrometry (GC-MS) Analysis of Aqueous extracts

*P. ostreatus* and *A. bisporus* aqueous extracts were frozen at −80 °C and powdered by drying in a lyophilizer. Then, chemical analysis was performed in the Central Laboratory of the Faculty of Postgraduate Studies for Advanced Sciences, Beni-Suef University, Egypt by using Gas Chromatography (GC) system 7890A/5975C Inert Mass Spectrometry (MS) with Triple Axis Detector, Ailent Technologies, Germany. Samples were injected in a splitless mode. The oven temperature program during analysis was as follows: 110 °C for 1 min, then 6 °C/min to 280 °C for 10 min. The equilibration time and the run time were 5 min and 39.333 min, respectively. The injection volume was 1 μL. Temperatures of the injector, MS source and MS Quad were 250 °C, 230 °C and 150 °C, respectively. Agilent 19091S-433: 2330.46415 HP-5MS 5% Phenyl Methyl Silox (30 m × 250 µm × 0.25 µm) column was used. Helium was used as a carrier at a flow rate of 1 mL/min. The constituents were recognized by comparing their mass spectra with the spectra of derivatives in the Library Search Report (C:\Database\NIST11.L).

The representation and interpretation of GC-MS results were revised by Prof. Dr. Sameh F. AbouZid, Pharmacognosy Department, Faculty of Pharmacy, Beni-Suef University, Beni-Suef, Egypt, Dr. Hassan M. Abdel-Aziz, Associate professor of Organic Chemistry, Department of Chemistry, Faculty of Science, Beni-Suef University, Egypt and Dr. Mai Raslan, Associate Professor of Biotechnology, Biotechnology and Life Sciences Department, Faculty of Postgraduate Studies for Advanced Sciences, Beni-Suef University, Egypt.

### 2.6. Preparation of Carvedilol Dose

The dose of carvedilol was prepared by dissolving 6 tablets of the drug (25 mg) in 25 mL distilled water (30 mg/5 mL) and was supplied to the rats by oral gavage at dose level of 30 mg (dissolved in 5 mL distilled water)/kg b.w. [49] daily for seven weeks. Carvedilol was obtained from Acapy Pharmaceutical Company, Egypt.

### 2.7. Induction of Hyperoxaluria

It has been shown by many researchers that a model of hyperoxaluria and urolithiasis in rats can be induced by ethylene glycol [50,51,52]. Hyperoxaluria, in the present study, was induced by adding ethylene glycol to the drinking water (0.75% *v*/*v*) [53,54] for 28 days. Ethylene glycol was purchased from S.D. Fine-Chem Limited, Mumbai, India.

### 2.8. Animal Grouping

After accommodation period, rats were allocated into five groups of six rats each as follow:

**Group 1 (normal control)** was supplied with tap water as a drinking water for nine weeks ad libitum for nine weeks and was given the equivalent volume of the vehicle (distilled water) 5 mL/kg b.w. daily by oral gavage during the last seven weeks.

**Group 2 (ethylene glycol group)** received ethylene glycol in drinking tap water (0.75% *v*/*v*) *ad libitum* for nine weeks and was supplemented with the equivalent volume of the vehicle (distilled water) 5 mL/kg b.w. daily by oral gavage during the last seven weeks.

**Group 3 (ethylene glycol and aqueous extract of *P. ostreatus* group)** received ethylene glycol in drinking tap water (0.75% *v*/*v*) ad libitum for nine weeks and was supplemented with the *P. ostreatus* aqueous extract by oral gavage at dose level of 100 mg (infused in 5 mL boiling distilled water)/kg b.w. daily during the last seven weeks.

**Group 4 (ethylene glycol and aqueous extract of*****A. bisporus*****group)** was given ethylene glycol in drinking tap water (0.75% *v*/*v*) ad libitum for nine weeks and was supplemented with the *A. bisporus* aqueous extract by oral gavage at dose level of 100 mg (infused in 5 mL boiling distilled water)/kg b.w. daily for the last seven weeks.

**Group 5 (ethylene glycol and carvedilol group)** was given ethylene glycol in drinking tap water (0.75% *v*/*v*) ad libitum for nine weeks and was supplemented with the carvedilol by oral gavage at dose level of 30 mg (dissolved in 5 mL distilled water)/kg b.w. daily during the last seven weeks.

Normal control (Group 1) was regarded as a control group for Group 2, while Group 2 was regarded as a control group for Groups 3–5.

### 2.9. Blood and Tissue Sampling

By the end of the experiment, rats in all groups were sacrificed under diethyl ether inhalation anesthesia after overnight fasting. For each rat, blood was obtained in clotting activated gel tubes from jugular vein. Then, the urine was collected from urinary bladder after dissection. The blood was centrifuged at 3000 round per minute (rpm) for 30 min. The sera were quickly aspirated into Eppendorf tubes for analysis of serum biomarkers of kidney functions. Specific gravity of urine from each rat was detected by Medi-Test Combi 10^®^ SGL strips obtained from Macherey-Nagel GmbH, Neumann-Neander-Str. 6-8 D52355 Duren, Germany. The obtained sera and urine samples were kept in deep freezer at −30 °C pending their use. Kidneys of each animal were excised, and then one kidney of each rat was fixed in 10% neutral buffer formalin for histopathological and immunohistochemical studies. A half gram of kidney of each animal was homogenized in 5 mL of 0.9% isotonic sterile saline (10% *w*/*v*) by Teflon homogenizer (Glas-Col, Terre Haute, IN, USA). The homogenates were centrifuged at 3000 rpm for 5 min and the homogenate supernatants were separated for determination of calcium and oxalate levels.

### 2.10. Biochemical Analysis

Serum and urine creatinine, urea and uric acid levels were determined by kits obtained from Diamond Diagnostics (Egypt) based on the methods of Murray [55], Tabacco et al. [56] and Fossati et al. [57], respectively. Serum, urine and kidney oxalate content were assayed based on the method of Young [58] by using kits from Ben-Biochemical Enterprise (Italy). Kidney calcium content was determined by using kits of Biodiagnostic (Egypt) based on the method of Gindler and King [59]. Fex Urea was calculated from formula: Fex Urea= [(urine urea/serum urea)/(urine creatinine/serum creatinine)] × 100 [60].

### 2.11. Determination of TNF-α and IL-1β Levels by ELISA

Serum TNF-α level was determined by rat TNFα ELISA kit purchased from MyBioSource, Inc., San Diego, CA 92195-3308 USA according manufacturer’s instructions. Serum IL-1β level was estimated by rat IL-1β ELISA kit purchased from Cloude-Clone Corp., Katy, TX 77494, USA according manufacturer’s instructions. 

### 2.12. Histological Investigations

The kidneys fixed in 10% neutral buffered formalin were sent to the Department of Pathology, National Cancer Institute, Cairo University, Egypt for processing, blocking, sectioning and staining with haematoxylin and eosin based on the method of Banchroft et al. [61]. The stained sections were examined by light microscope to detect the histological changes.

### 2.13. Immunohistochemical Investigations

For immunhistochemical investigations, kidney sections (4 µm thick) were mounted onto positive-charged slides (Fisher Scientific, Pittsburgh, PA, USA) to detect NF-κB, p53 and Bcl-2. The NF-κB, Bcl-2 and p53 immunoreactivity were determined following Gao and Zhou [62]. Briefly, before the incubation with antibodies, endogenous peroxidase activity was quenched, and slides were washed and then incubated in a blocking solution of hydrogen peroxide 1% in methanol in darkness for 15 min. After antigen retrieval, sections were rinsed in tap water and then phosphate buffer saline. Primary rat antibody for NF-κB (Thermo Fisher Scientific, Waltham, MA 02451, USA) and rat antibodies for p53 and Bcl-2 (DakoCytomation, Carpinteria, CA, USA) diluted 1:100 in PBS, were applied for 1 h at 37 °C. Secondary biotinylated antibody diluted 1:100 in PBS was applied for a period of 30 min at 37 °C. Streptavidinbiotin or avidin-biotin peroxidase (ABC/HRP) was applied for 10 min at room temperature. Bound antibody complex was visualized by the reaction of 3, 3′-diaminobenzidine (DAB) substrate and counter stained with haematoxylin.

### 2.14. Imaging and Semi-Quantitative Analysis of NF-κB, p53 and Bcl-2 Expressions

The yellowish brown colored stained in percent area was detected by ImageJ software, US National Institutes of Health, Bethesda, Maryland, USA (http://imagej.nih.gov/ij/).

### 2.15. Western Blot Analysis

The ReadyPrepTM protein extraction kit (total protein) obtained from Bio-Rad Inc. (Catalog #163-2086) was employed according to manufacturer’s instructions. Bradford Protein Assay Kit (SK3041) for quantitative protein analysis was provided by Bio basic Inc. (Markham, Ontario, L3R 8T4, Canada) according to manufacturer’s instructions. Protein in each sample was loaded and separated by using TGX Stain-Free™ FastCast™ Acrylamide Kit (SDS-PAGE), provided by Bio-Rad Laboratories Inc. (Cat # 161-0181), according to manufacturer’s instructions. The protein bands separated by polyacrylamide gel electrophoresis were transferred from gel to PDVF (polyvinylidene difluoride) membrane using BioRad Trans-Blot Turbo. Tris-buffered saline with Tween 20 (TBST) buffer and 3% bovine serum albumin (BSA) was used to block the membrane at room temperature for 1 h. The 1^ry^ antibodies NF-κB p65, NF-κB and Bax purchased from Santa Cruz Biotechnology, Inc. (USA) as well as Bak purchased from Aviva Systems Biology (7700 Ronson Road, Ste 100, San Diego, CA 92111 USA) were diluted in TBST according to manufactured instructions. Incubation was done overnight for each 1^ry^ antibody solution, against the blotted target protein, at 4 °C. The blot was rinsed 3–5 times for 5 min with TBST. Incubation was done for the horse raddish peroxidase (HRP)-conjugated 2^ry^ antibody (Goat anti-rabbit IgG – HRP – 1 mg Goat MAb; Novus Biologicals) solution against the blotted target protein for 1 h at room temperature. Then, the blot was rinsed 3–5 times for 5 min with TBST. The chemiluminescent substrate (ClarityTM Western ECL substrate Bio-Rad cat#170-5060) was applied to the blot based on the manufacturer’s recommendation. Briefly, equal volumes were added from both solution A (Clarity Western luminal/enhancer solution) and solution B (peroxidase solution). The chemiluminescent signals were captured using a CCD (charge coupled device) camera-based imager. Image analysis software was used to read the band intensity of the target proteins against control sample β-actin (housekeeping protein) by protein normalization on the ChemiDoc MP imager.

### 2.16. Statistical Analysis

The data were analyzed using the one-way analysis of variance (ANOVA) by PC-STAT program [63] followed by LSD test to compare different groups with each other. Data were expressed as mean ± standard error means (SEM). F-probability obtained from one-way ANOVA expresses the general effect on each parameter between groups throughout the experiment.

## 3. Results

### 3.1. Phytochemical Screening

Phytochemical analysis (Table 1) of *P. ostreatus* and *A. bisporus* indicated the presence of carbohydrates (or glycosides), alkaloids, flavonoids, resins, tannins and unsaturated sterols.

### 3.2. GC-MS Analysis of P. ostreatus Aqueous Extract

**Figure 2:**
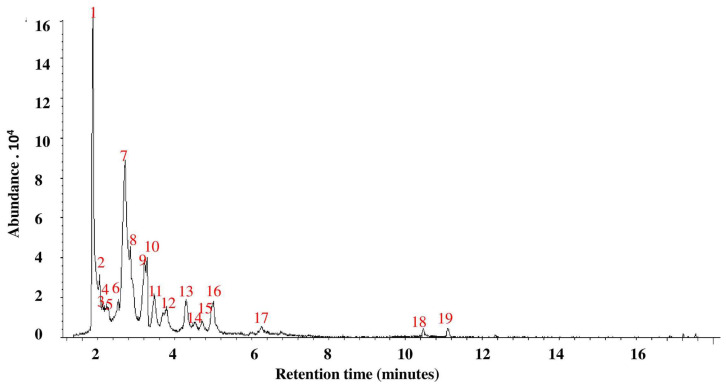
GC-MS analysis of *P. ostreatus* aqueous extract.

The GC-MS analysis of *P. ostreatus* aqueous extract (Table 2, Figure 2 and Figure S2) indicated the identification of nineteen phytochemicals. The most abundant constituents were *N*-hydroxy-*N*-methyl methenamine (24.2%) and (S)-(+)-isoleucinol (23.0%).

### 3.3. GC-MS Analysis of A. bisporus Aqueous Extract

**Figure 3:**
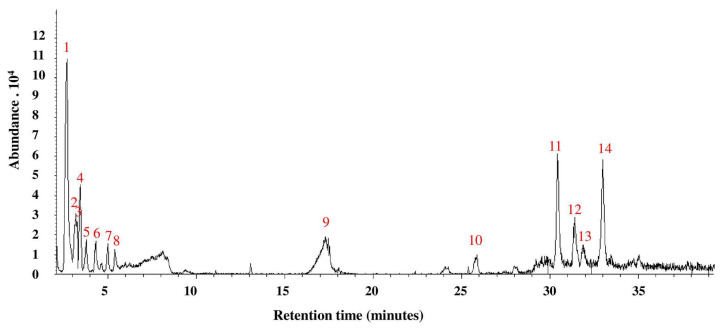
GC-MS analysis of *A. bisporus* aqueous extract.

The GC-MS analysis of *A. bisporus* aqueous extract (Table 3, Figure 3 and Figure S3) allowed the identification of fourteen different phytochemicals. The most abundant constituents include N-methoxy-methanamine (30.6%), 6-ethyl-2,3-dihydro-2,7-dimethyl-5-oxo-5H-oxazolo[3,2-a]pyridine-8-carbonitrile (13.7%) and Fumaric acid, 2-heptyl octyl ester (13.11%).

### 3.4. Effect on Body Weight, Kidney Weight and Relative Kidney Weight

**Figure 4:**
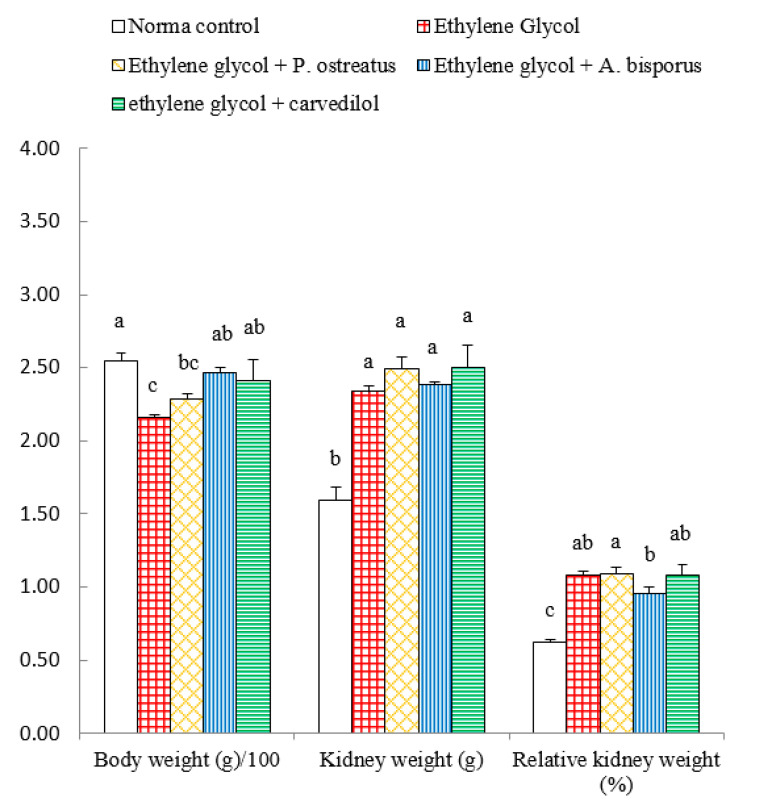
Effect of *P. ostreatus* and *A. bisporus* aqueous extracts and carvedilol on body weight, kidney weight and relative kidney weight of ethylene glycol-administered rats. For each parameter, means which share the same letter(s) are not significantly different.

Administration of ethylene glycol to Wistar rats induced a highly significant decrease (*p* < 0.01; LSD) in body weight, by 15.29%, as compared with normal control. The treatment of ethylene glycol-induced hyperoxaluric rats with *A. bisporus* infusion and carvedilol induced a significant increase (*p* < 0.05; LSD) while the treatment with *P. ostreatus* infusion produced a non-significant increase (*p* > 0.05; LSD). On the other hand, the kidney weight and relative kidney weight significantly increased (*p* < 0.01; LSD) in ethylene glycol-induced hyperoxaluric rats recording changes of 46.92% and 93.91%, respectively, as compared with the normal control. Meanwhile, the treatment of hyperoxaluric rats with mushroom infusions and carvedilol has no significant effect (*p* > 0.05; LSD) on kidney weight and relative kidney weight as compared with the ethylene glycol-induced hyperxaluric Wistar rats (Figure 4 and Figure S4).

### 3.5. Effect on Serum Creatinine, Urea and Uric Acid Levels

**Figure 5:**
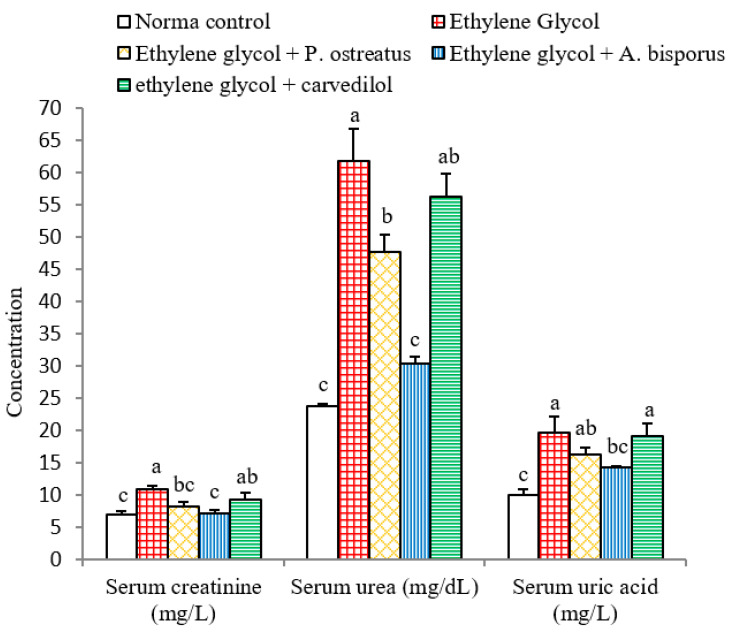
Effect of *P. ostreatus* and *A. bisporus* aqueous extracts and carvedilol on levels of serum parameters related to kidney function of ethylene glycol-administered rats. For each parameter, means which share the same letter(s) are not significantly different.

Administration of ethylene glycol to Wistar rats induced a highly significant increase (*p* < 0.01; LSD) in serum creatinine, urea and uric acid levels by 53.52%, 159.97% and 95.05%, respectively, as compared with normal control. The treatment of ethylene glycol-induced hyperoxaluric rats with *P. ostreatus* and *A. bisporus* infusions, meanwhile, induced a highly significant decrease (*p* < 0.01; LSD) in creatinine and urea levels. The uric acid level was non-significantly (*p* > 0.05; −17.26%) and significantly (*p* < 0.05; −26.90%) decreased as a result of treatment with *P. ostreatus* and *A. bisporus*, respectively, while treatment with carvedilol caused a non-significant (*p* > 0.05; LSD) decrease of all above parameters (Figure 5 and Figure S5). Thus, *A. bisporus* extract followed by *P. ostreatus* extract seemed to be more effective in reducing elevated serum creatinine, urea and uric acid levels in hyperoxaluric rats.

### 3.6. Effect on Urine Creatinine, Urea and Uric Acid Levels

**Figure 6:**
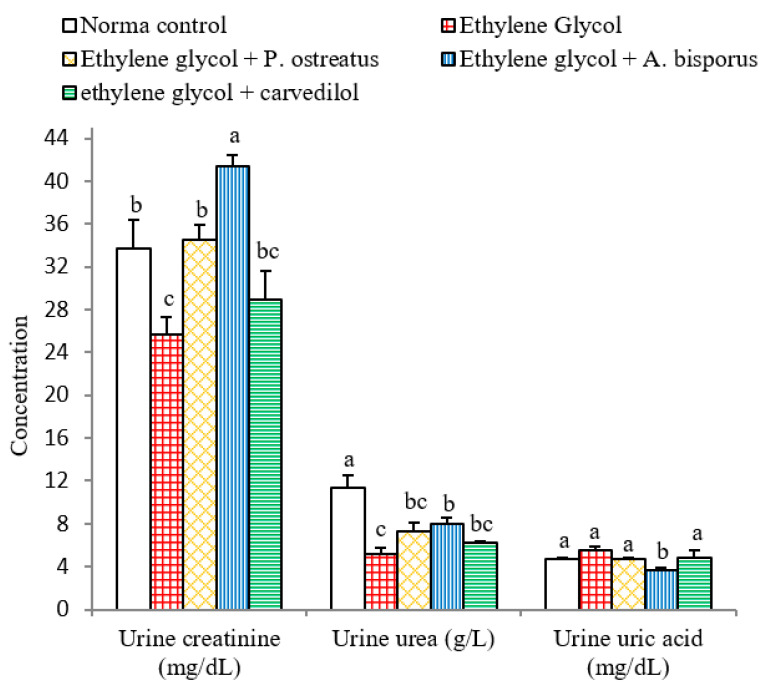
Effect of *P. ostreatus* and *A. bisporus* aqueous extracts and carvedilol on different urine parameters related to renal efficiency of ethylene glycol-administered rats. For each parameter, means which share the same letter(s) are not significantly different.

After administration of ethylene glycol to albino rats, urine creatinine and urea levels reduced very significantly (*p* < 0.01; LSD) while, urine uric acid level was non-significantly affected (*p* > 0.05; LSD) compared to the normal control. Treatment with *P. ostreatus* and *A. bisporus*, however, led to a highly significant increase (*p* < 0.01; LSD) of urine creatinine level, while urine urea was non-significantly (*p* > 0.05; 40.08%) and significantly (*p* < 0.05; 53.18%) increased as a result of treatment with *P. ostreatus* and *A. bisporus*, respectively. On the other hand, urine uric acid level decreased non-significantly (*p* > 0.05; −15.49%) with *P. ostreatus* and highly significant (*p* < 0.01; −34.41%) with *A. bisporus*. In contrast, treatment with carvedilol resulted in only non-significant alterations (*p* > 0.05; LSD) in all of the above parameters compared with ethylene glycol control rats (Figure 6 and Figure S6). Overall, therefore, *A. bisporus* followed by *P. ostreatus* appeared to be more effective in increasing the urine creatinine and urea levels and decreasing urine uric acid levels than carvedilol.

### 3.7. Effect on Various Ratios Related to Kidney Functions:

**Figure 7:**
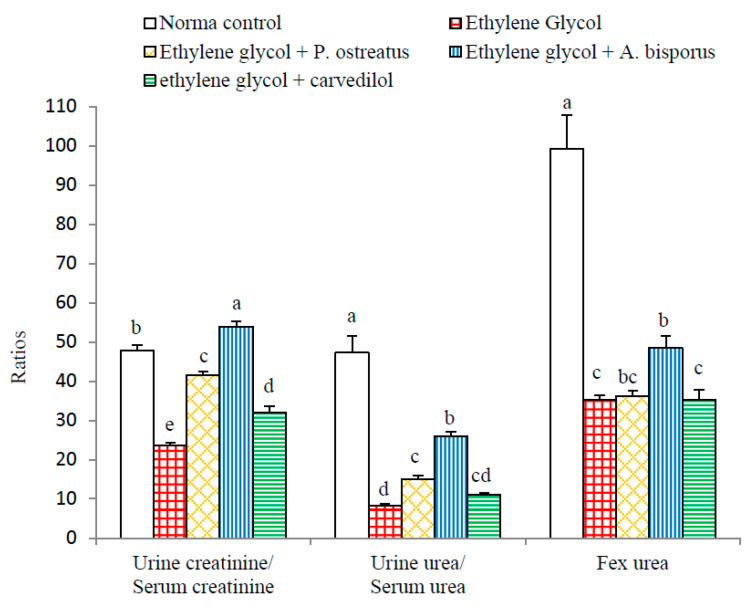
Effect of *P. ostreatus* and *A. bisporus* aqueous extracts and carvedilol on various ratios related to kidney function of ethylene glycol-administered rats. For each parameter, means which share the same letter(s) are not significantly different.

The effects on various ratios related to kidney functions are depicted in Figure 7 and Figure S7. Highly significant reductions (*p* < 0.01; LSD) in urine creatinine/serum creatinine, urine urea/ serum urea and Fex Urea ratios were induced as a result of administering ethylene glycol to albino rats recording changes of −50.59%, −82.41% and −64.55%, respectively, as compared to the normal control.

The treatment of ethylene glycol-induced urolithic rats with all the tested agents, on the other hand, led to highly significant increases (*p* < 0.01; LSD) in the urine creatinine/serum creatinine ratio. With respect to the urine urea/serum urea ratio, significant (*p* < 0.05; 80.17%), highly significant (*p* < 0.01; 211.66%) and non-significant (*p* > 0.05; 33.77%) increases were attained following the treatment with *P. ostreatus*, *A. bisporus* and carvedilol, respectively. Fex Urea was non-significantly increased (*p* > 0.05; LSD) after treatment with *P. ostreatus* and carvedilol and significantly increased (*p* < 0.05; 37.69%) after treatment with *A. bisporus*.

### 3.8. Effect on Serum, Urine and kidney Oxalate Levels

**Figure 8:**
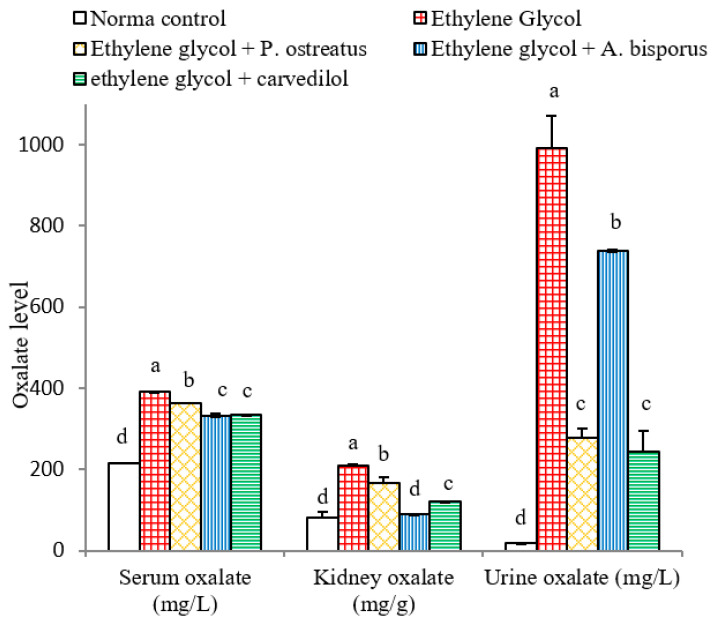
Effect of *P. ostreatus* and *A. bisporus* aqueous extracts and carvedilol on serum, kidney and urine oxalate levels in ethylene glycol-administered rats. For each parameter, means which share the same letter(s) are not significantly different.

Serum, urine and kidney oxalate levels were highly significantly increased (*p* < 0.01; LSD) as a result of administration of ethylene glycol to albino rats recording changes of 81.39%, 5446.93% and 155.75%, respectively, as compared to the normal control. The treatment of ethylene glycol-induced urolithic rats with *P. ostreatus*, *A. bisporus* and carvedilol, however, induced a decline in the serum oxalate level, which was significant with *P. ostreatus* and highly significant with *A. bisporus* and carvedilol. On the other hand, a highly significant (*p* < 0.01; LSD) decrease in urine and kidney oxalate levels was produced as a result of treatment with the mushroom infusions and carvedilol (Figure 8 and Figure S8). Overall, *A. bisporus* extract was the most effective in decreasing kidney oxalate level while carvedilol was the most effective in decreasing urine oxalate.

### 3.9. Effect on Urine Specific Gravity

**Figure 9:**
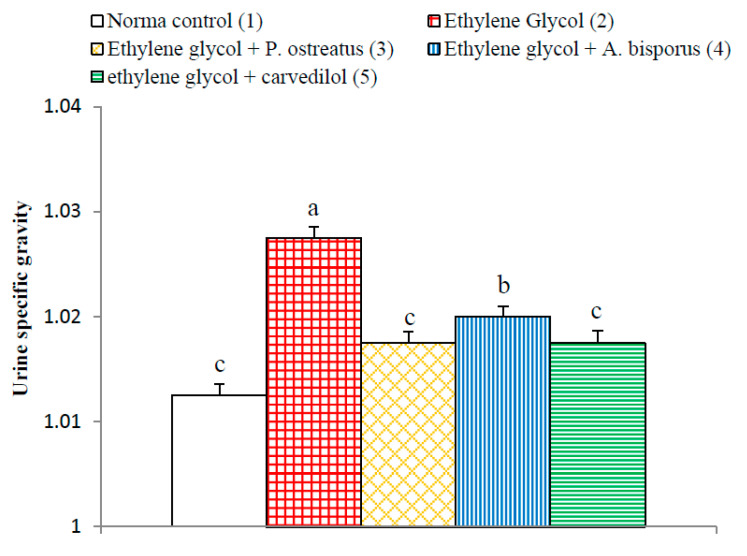
Effect of *P. ostreatus* and *A. bisporus* aqueous extracts and carvedilol on urine specific gravity in ethylene glycol-administered rats. Means which share the same letter(s) are not significantly different.

The urine specific gravity exhibited similar behavioral pattern as urine oxalate level. The ethylene glycol-induced hyperoxaluric rats exhibited a significant increase (*p* < 0.01; LSD) in urine specific gravity as compared to normal control group. The treatment of ethylene glycol-induced with *P. ostreatus* and *A. bisporus* aqueous extracts and carvedilol produced a significant decrease (*p* < 0.01; LSD) of the elevated urine hyperoxaluric rats with specific gravity (Figure 9 and Figure S9). *P. ostreatus* aqueous extract and carvedilol was more effective than *A. bisporus* aqueous extract in decreasing the elevated urine specific gravity.

### 3.10. Effect on Kidney Calcium Level

**Figure 10:**
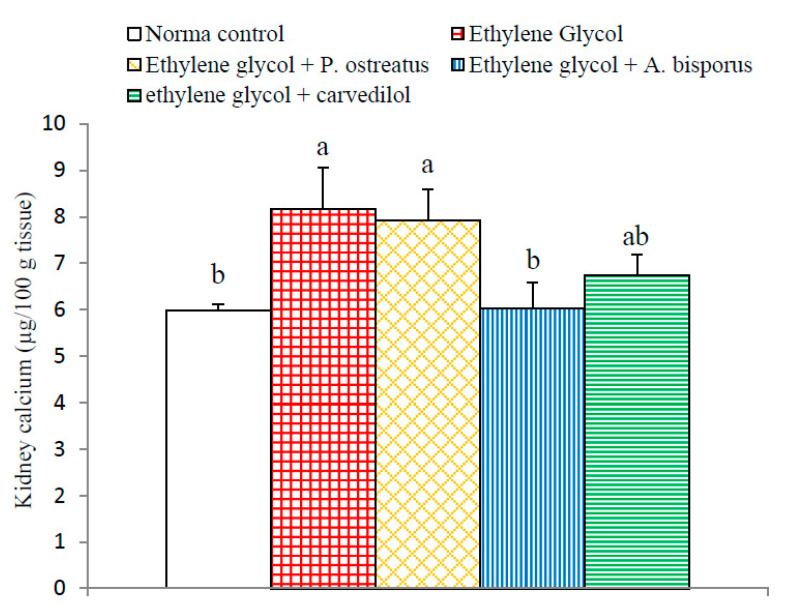
Effect of *P. ostreatus* and *A. bisporus* aqueous extracts and carvedilol on kidney calcium content in ethylene glycol-administered rats. Means which share the same letter(s) are not significantly different.

Administration of ethylene glycol induced a significant increase (*p* < 0.05; 36.45%) in kidney calcium level compared to the normal control. Kidney calcium levels were significantly decreased as a result of treatment with *A. bisporus* (*p* < 0.05; −25.98%) and non-significantly decreased following treatment with *P. ostreatus* (*p* > 0.05; −2.94%) and carvedilol (*p* > 0.05; −17.28%) as compared with the urolithic control group (Figure 10 and Figure S10). Thus, *A. bisporus* infusion seemed to be the most potent in decreasing the elevated kidney calcium and oxalate content in ethylene glycol-induced hyperoxaluric rats.

### 3.11. Effects on Serum TNF-α and IL-1β Levels

**Figure 11:**
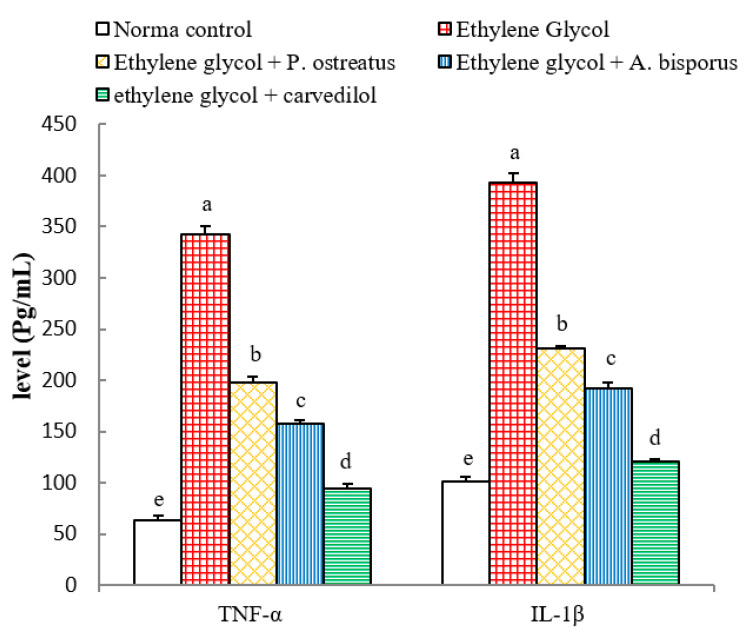
Effect of *P. ostreatus* and *A. bisporus* aqueous extracts and carvedilol on serum TNF-α and IL-1β levels in ethylene glycol-administered rats. Means which share the same letter(s) are not significantly different.

The ethylene glycol-induced hyperoxaluric rats exhibited a highly significant elevation (*p* < 0.01; LSD) in serum TNF-α and IL-1β levels, with changes of 437.25% and 285.75% respectively, as compared with normal control. The treatment of the ethylene glycol-induced hyperoxaluric rats with *P. ostreatus* and *A. bisporus* aqueous extracts and carvedilol induced a highly significant decrease (*p* < 0.01; LSD) of the elevated serum TNF-α and IL-1β levels; carvedilol was the most potent (Figure 11 and Figure S11).

### 3.12. Histological Effects

**Figure 12:**
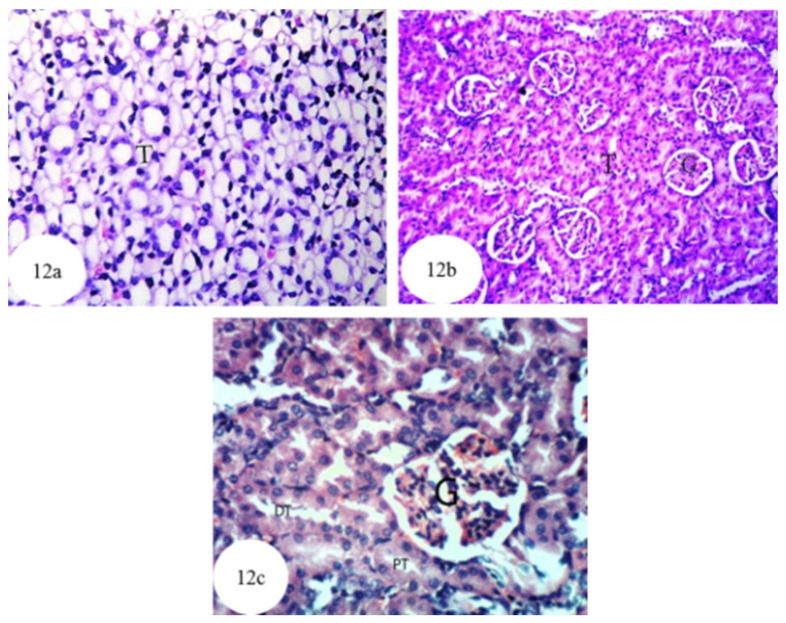
Photomicrographs of haematoxylin and eosin stained sections of kidney of a normal control rat showing normal histological structure of the medullary portion and the tubules (T) (**a**) (×256), normal histological structure of the cortex portion containing glomerulus (G) and tubule (T) (**b**) (×128) and normal histological structure of the renal parenchyma where glomerulus (G), proximal tubule (PT) and distal tubule (DT) were observed (**c**) (×400).

Microscopical examination of kidney sections of normal rats (Figure 12a–c and Figure S12) revealed the tissue to have a normal histological structure. The kidney is divided into an inner region, the medulla and an outer region, the cortex. The medulla consists of collecting tubules and thin segments comprising the loop of Henle. The cortex, meanwhile, contains glomeruli and tubules.

**Figure 13:**
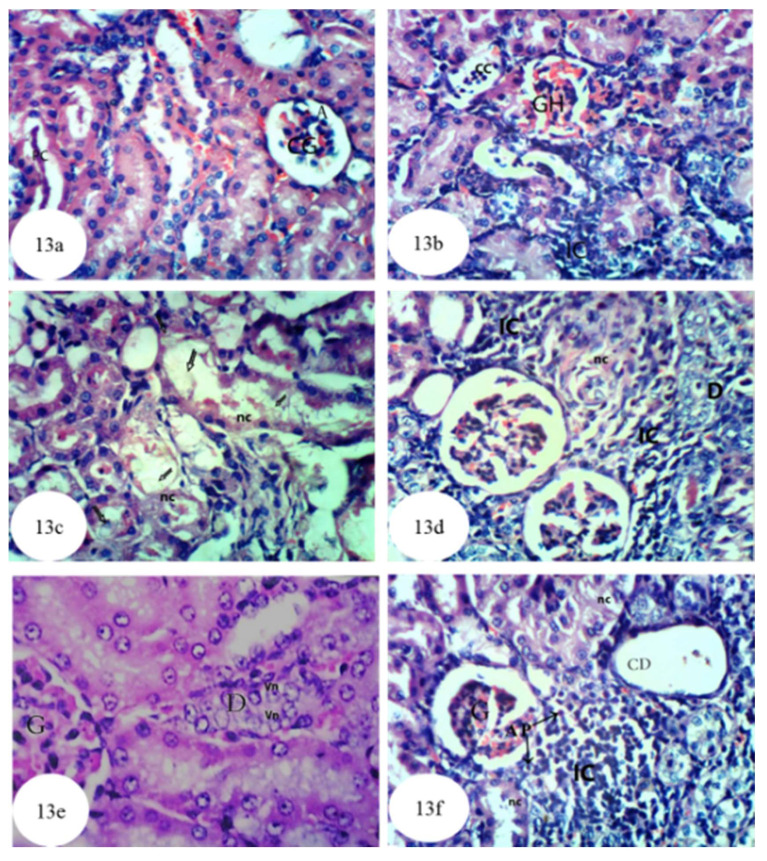
Photomicrographs of hematoxylin and eosin stained sections of kidney of ethylene glycol control rat showing atrophy (A) and congestion (CG) of the glomerular tufts and presence of protein casts (PC) in the lumen of the renal tubules (**a**) (×400); interstitial nephritis marked by inflammatory cell (IC) infiltration and cellular casts (CC) in the lumen of the renal tubules and glomerular hemorrhage (GH) (**b**) (×400); necrosis (nc) of renal tubules and presence of oxalate crystals (arrows) in the lumen of the renal tubules (**c**) (×400); dysplastic tubular cells (D) and focal necrosis (nc) of the renal tubules associated with massive inflammatory cells (IC) infiltration (**d**) (×400); enlarged vesicular nuclei (vn) with irregular arrangement (*dysplasia* and *anaplasia*) (D) in the lining epithelium of the renal tubules (**e**) (×256); and cystic dilatation (CD) of the renal tubules, focal inflammatory cells (IC) infiltration, necrosis (nc) and apoptotic cells (AP) (**f**) (×400).

In rats administered with ethylene glycol, atrophy and congestion were observed in the glomerular tufts, and the lumina of the renal tubules were affected by protein casts (Figure 13a and Figure S13), cellular casts, interstitial nephritis, glomerular hemorrhages (Figure 13b), necrosis and dense oxalate crystals (Figure 13c). In addition, as shown in Figure 13d, the kidney tissues exhibited dysplastic tubular cells and focal necrosis associated with massive infiltration of inflammatory cells within the renal tubules, as well as enlarged vesicular nuclei with an irregular arrangement (dysplasia and anaplasia) in the lining of the epithelium of some renal tubules (Figure 13e), and infiltration of focal inflammatory cells, necrosis, cystic dilatation and apoptotic cells (AP) in other renal tubules (Figure 13f).

**Figure 14:**
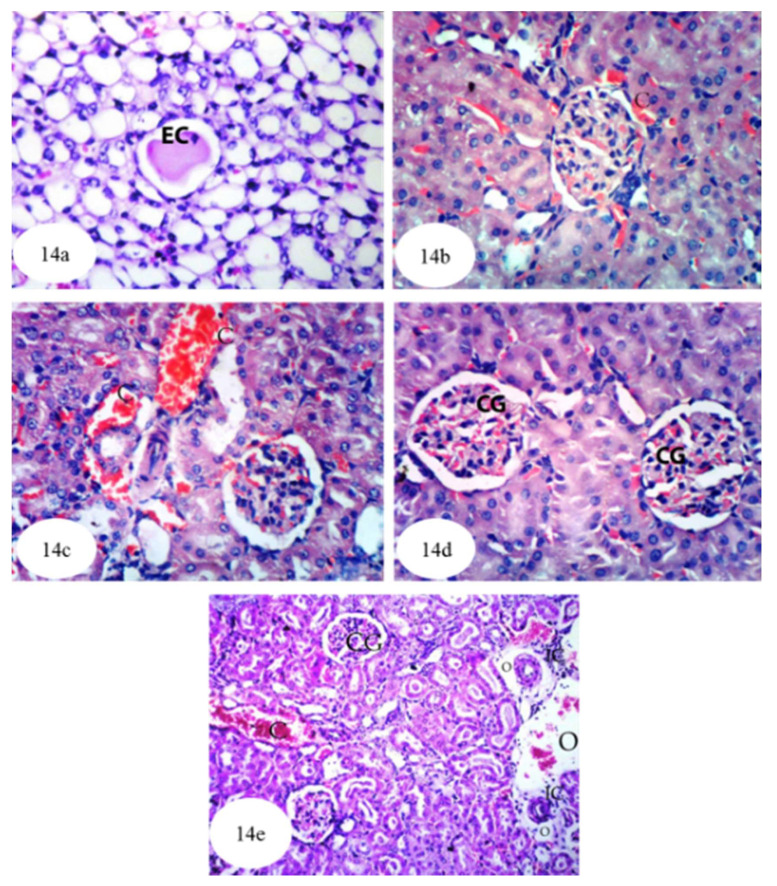
Photomicrographs of hematoxylin and eosin stained sections of kidney of ethylene glycol-administered rat treated with *P. ostreatus* aqueous extract showing homogenous eosinophilic casts (EC) in the lumen of few medullary tubules (**a**) (×256), slight congestion (C) of intertubular blood capillaries (**b**) (×400); congestion (C) of intertubular blood vessles (**c**) (×400); slight congestion of the glomrular tufts (CG) (**d**) (×400); and congestion (C) in the cortical blood vessels and glomeruli (CG) with perivascular inflammatory cells (IC) infiltration and oedema (O) (**e**) (×128).

As shown in Figure 14 and Figure S14, once ethylene glycol-administered rats were treated with *P. ostreatus*, homogenous eosinophilic casts were evident in the lumina of a few medullary tubules (Figure 14a). Moreover, slight congestion was observed in intertubular renal blood capillaries (Figure 14b,c) and in the glomerular tufts (Figure 14d). The congestion observed in the cortical blood vessels and the glomeruli was associated with oedema and infiltration of inflammatory cells into the perivascular tissue (Figure 14e). Despite these histological changes, the kidney architecture was markedly improved compared to that of ethylene glycol-administered control rats.

**Figure 15:**
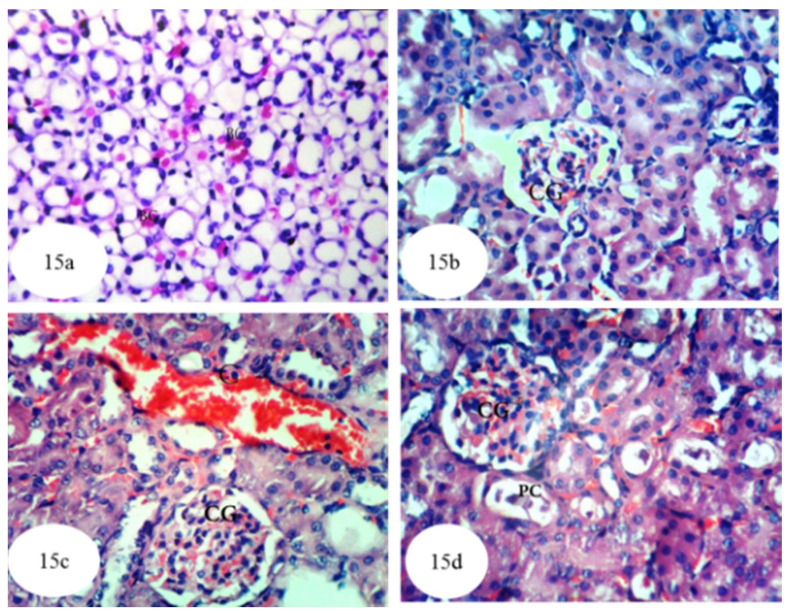
Photomicrographs of hematoxylin and eosin stained sections of kidney of ethylene glycol-administered rat treated with *A. bisporus* aqueous extract showing few red blood cells (BC) in the tubular lumen and in between the tubules at the medulla (**a**) (×256); normal renal structure with slight glomerular congestion (CG) (**b**) (×400); congestion (C) of renal blood vessels and glomeruli (CG) (**c**) (×400); and small protein casts (PC) in the lumen of some renal tubules and mild congestion in the glomeruli (CG) (**d**) (×400).

Figure 15 and Figure S15 show the histological results of treatment of ethylene glycol-administered rats with *A. bisporus*, revealing a considerable improvement in the kidney architecture. In that context, Figure 15a shows presence of red blood cells in and between the tubules at the medulla, while Figure 15b displays normal renal parenchyma with slight glomerular congestion. Figure 15c illustrates some congestion of renal blood vessels and glomeruli and Figure 15d shows the presence of small protein casts in the lumina of some renal tubules associated with mild congestion in the glomeruli.

**Figure 16:**
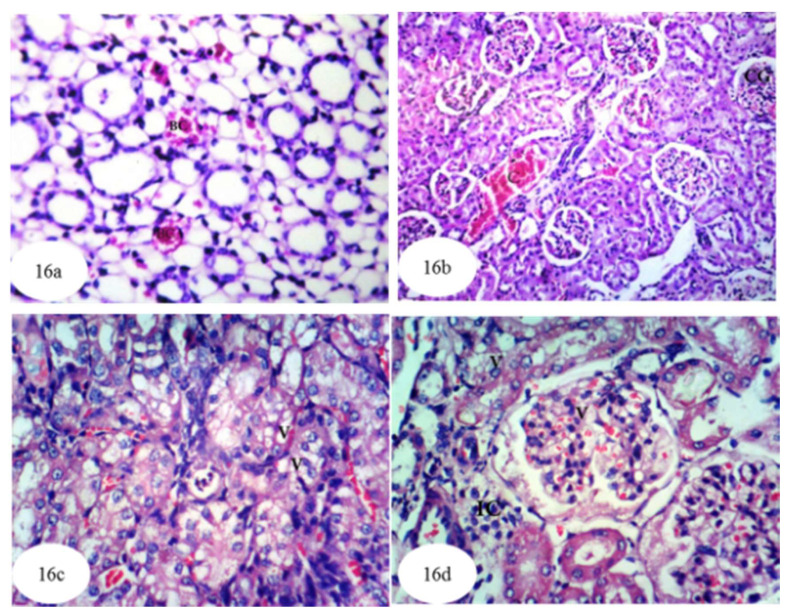
Photomicrographs of hematoxylin and eosin stained sections of kidney of ethylene glycol-administered rat treated with carvedilol showing red blood cells (BC) in some of the tubular lumen at the medulla (**a**) (×256); slight congestion (C) in the cortical blood vessels and glomeruli (CG) (**b**) (×128); vacuolization (V) of epithelial lining the tubules (**c**) (×400); and hypertrophy and vacuolization (V) of the glomerular tufts, vacuolization of epithelial lining renal tubules and perivascular inflammatory cells (IC) infiltration (**d**) (×400).

Figure 16 and Figure S16 shows the histological results of treatment of ethylene glycol-administered rats with carvedilol. Here, Figure 16a manifests the presence of red blood cells in the lumina of some medullary tubules, while Figure 16b reveals slight congestion in the cortical blood vessels and glomeruli. Figure 16c shows vacuolization of the epithelium lining the tubules while Figure 16d demonstrates, in addition to the latter change, hypertrophy and vacuolization of the glomerular tufts associated with infiltration of inflammatory cells into the perivascular tissue. Overall, the group treated with carvedilol showed noticeable amelioration of many the histological changes originally apparent after the administration of ethylene glycol.

### 3.13. Effect on Immunohistochemically Detected Kidney NF-κB, p53 and Bcl-2

**Figure 17:**
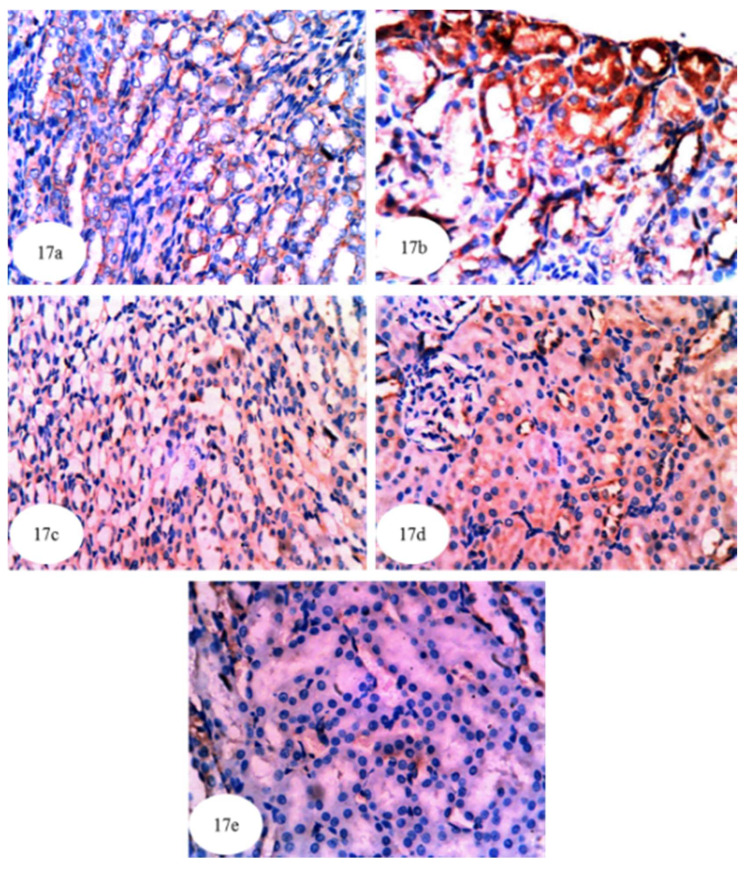
Photomicrographs (**a**–**e**) of immunohistochemically stained kidney sections. Images showed the higher amount of NF-κB (yellowish brown color) in cytoplasm and nuclei of ethylene glycol control group (**b**) (×384) as compared with normal control rats (**a**) (×384). The treatment of ethylene glycol administered rats with *P. ostreatus* (**c**) (×384) and *A. bisporus* (**d**) (×384) infusions and carvedilol (**e**) (×384) markedly decreased the dark brownish color of NF-κB.

**Figure 18:**
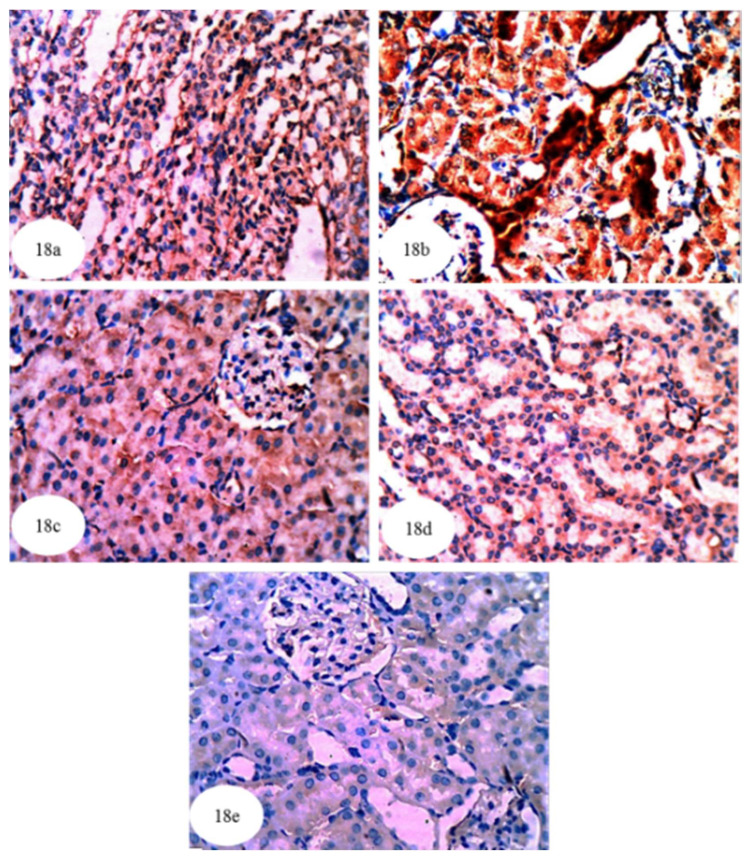
Photomicrographs (**a**–**e**) of immunohistochemically stained kidney sections showing the greater amount of the apoptotic marker p53 (yellowish brown color) in the cytoplasm and nuclei of the hyperoxaluric control rats (**b**) (×384) as compared with normal control group (**a**) (×384). The p53 was remarkably decreased after treatment of ethylene glycol-administered rats with *P. ostreatus* (**c**) (×384) and *A. bisporus* (**d**) (×384) infusions and carvedilol (**e**) (×384), which was indicated by decline of the intensity of yellowish brown color.

**Figure 19:**
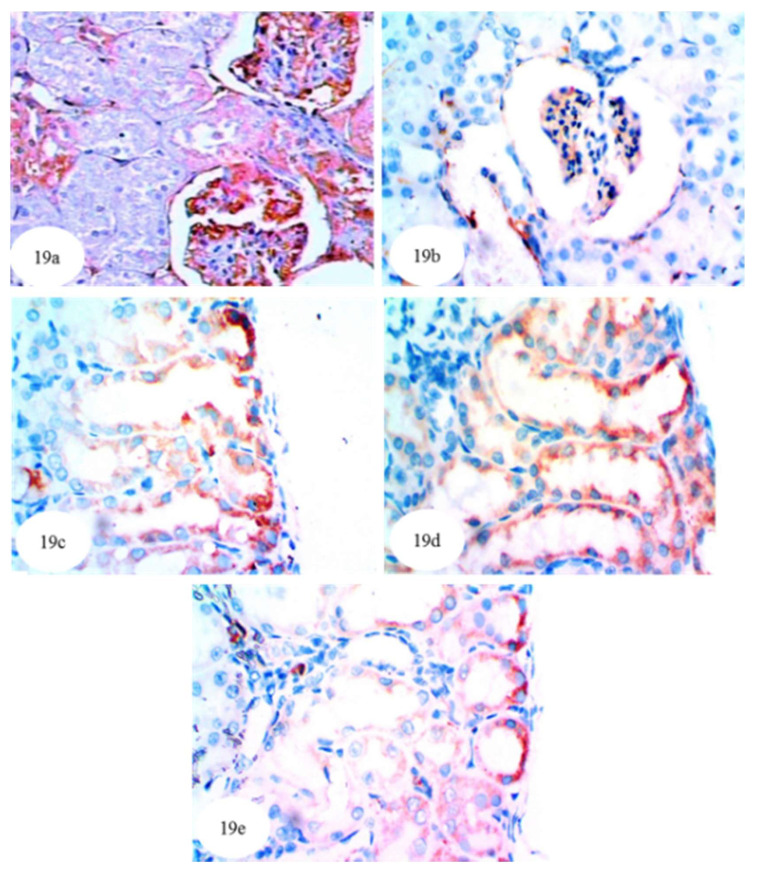
Photomicrographs of immunohistochemically stained kidney sections showing the lighter amount of the antiapoptotic marker Bcl-2 (yellowish brown color) in the cytoplasm of the urolithic rats (**b**) (×512) as compared with normal control group (**a**) (×512). Bcl-2 was potentially increased after treatment of ethylene glycol-administered rats with *P. ostreatus* (**c**) (×512), *A. bisporus* (**d**) (×512) and carvedilol (**e**) (×512), which was indicated by increase of intensity of the yellowish brown color.

In the present study, immunohistochemical staining was used to detect the expression of NF-κB, p53 and Bcl-2 in kidney tissues of the albino rats. NF-κB expression in cytoplasm and nuclei (illustrated by a yellowish brown colored staining) was noticeably increased in the kidneys of ethylene glycol control rats (Figure 17b and Figure S17) compared to normal rats (Figure 17a). The treatment of ethylene glycol-induced hyperoxaluric rats with extracts of *P. ostreatus* and *A. bisporus* and with carvedilol decreased the expression of NF-κB (Figure 17c–e); *P. ostreatus* and *A. bisporus* extracts were more effective than carvedilol. Similarly, p53 protein concentration (yellowish brown color) was remarkably increased in the cytoplasm and nuclei of the ethylene glycol control group (Figure 18b and Figure S18) as compared with normal rats (Figure 18a). Again, however, *P. ostreatus*, *A. bisporus* and carvedilol treatments all markedly reduced the p53 expression; *P. ostreatus* extract and carvedilol were more effective than *A. bisporus* extract (Figure 18–e). With respect to the expression of Bcl-2 (yellowish brown color) in the cytoplasm of the ethylene glycol rats, Figure 19b indicates a decrease as compared with normal rats (Figure 19a and Figure S19). Treatment of the ethylene glycol-induced urolithic rats with *P. ostreatus* and *A. bisporus* extracts and carvedilol appeared to lead to a considerable increase in the expression of Bcl-2; *P. ostreatus* and *A. bisporus* extracts were more effective than carvedilol (Figure 19c–e).

**Figure 20:**
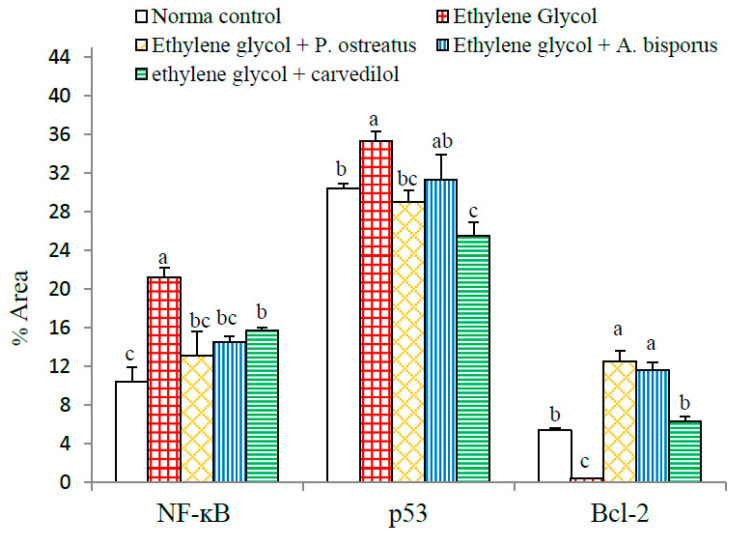
Effect of *P. ostreatus* and *A. bisporus* aqueous extracts and carvedilol on percent area of yellowish brown color of NF-κB, p53 and Bcl-2 expressions detected by image analysis of immunohistochemically stained kidney sections in ethylene glycol-administered rats. For each mediator, three kidney sections from three rats of each group were immunohistochemically stained and were subjected to ImageJ analysis. For each parameter, means which share the same superscript letter(s) are not significantly different.

With respect to statistical analysis of ImageJ results of immunohistochemical stained sections, ethylene glycol administration was found to be associated with highly significant increases in the expressions of NF-κB (*p* < 0.01; 103.55%) and a significant (*p* < 0.05; 16.08%) increase in p53 in the kidney compared to the normal control while the expression of Bcl-2 reduced highly significantly (*p* > 0.05; −92.42%). When the ethylene glycol-administered rats were supplemented with mushroom extracts, there was a highly significant (*p* < 0.01; LSD) decrease in the expression of NF-κB but only a significant (*p* < 0.05; LSD) decrease in NF-κB expression following treatment with carvedilol. When ethylene glycol-administered rats were treated with *P. ostreatus*, the expression of p53 was highly significantly reduced by 17.87% (*p* < 0.01; LSD) while treatment with carvedilol led to a highly significant reduction of 27.65%. Treatment with *A. bisporus*, however, led to a non-significant (*p* > 0.05) reduction of 11.44% in p53 expression. All treatment agents, however, were associated with a highly significant increase (*p* < 0.01; LSD) in Bcl-2 expression when they were administered to rats previously treated with ethylene glycol (compared to the urolithic control group) (Figure 20 and Figure S20).

### 3.14. Effect on Kidney p65, p50, Bax and Bak Detected by Western Blot

**Figure 21:**
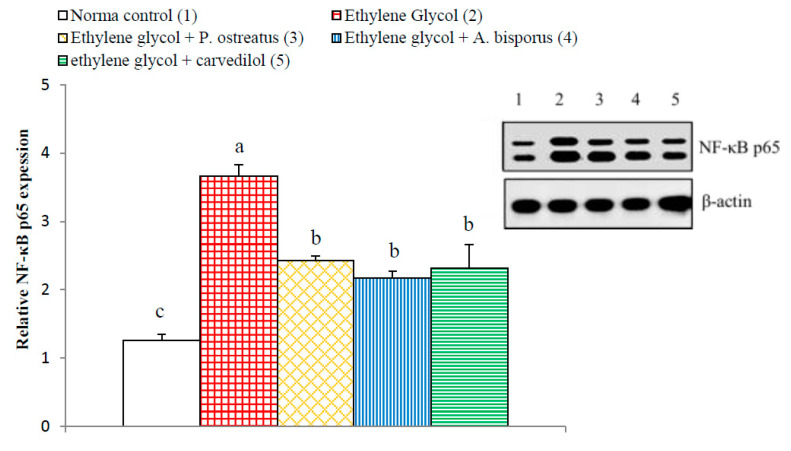
Effect of *P. ostreatus* and *A. bisporus* aqueous extracts and carvedilol on relative kidney NF-κB p65 expression detected by Western blot in ethylene glycol-administered rats. Means which share the same letter(s) are not significantly different.

**Figure 22:**
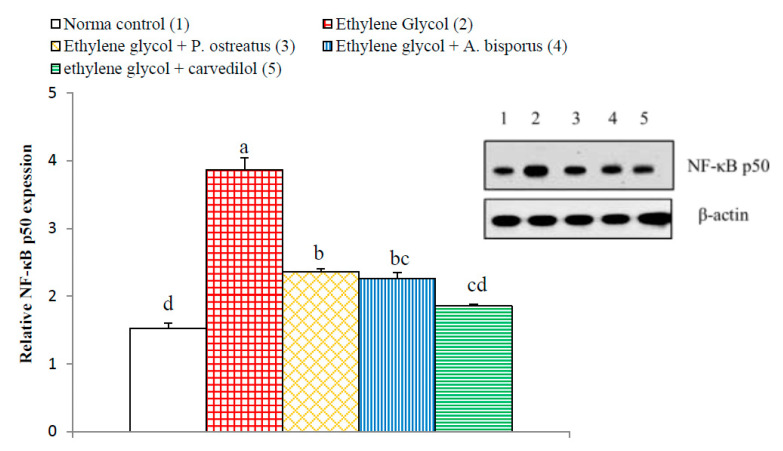
Effect of *P. ostreatus* and *A. bisporus* aqueous extracts and carvedilol on relative kidney NF-κB p50 expression detected by Western blot in ethylene glycol-administered rats. Means which share the same letter(s) are not significantly different.

The kidney NF-κB p65 and NF-κB p50 significantly increased (*p* < 0.01; LSD) in ethylene glycol-induced hyperoxaluric rats, by 190.48% and 153.04%, respectively, as compared with normal control. Meanwhile, the treatment of hyperoxaluric rats with *P. ostreatus* and *A. bisporus* aqueous extracts and carvedilol successfully counteracted the ethylene glycol-induced elevations in NF-κB p65 and NF-κB p50, producing a highly significant decrease (*p* < 0.01; LSD) as compared with the ethylene glycol-induced hyperoxaluric control rats. The effect of carvedilol was most potent in decreasing the elevated NF-κB p50 level (Figure 21 and Figure 22, Figures S21 and S22).

**Figure 23:**
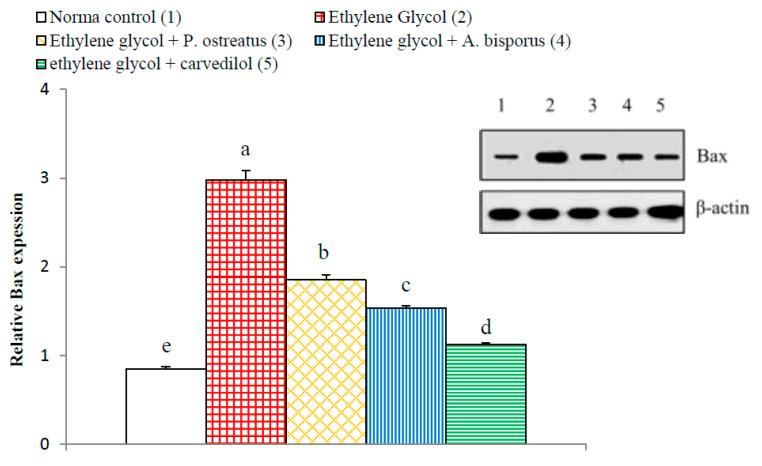
Effect of *P. ostreatus* and *A. bisporus* aqueous extracts and carvedilol on relative kidney Bax expression detected by Western blot in ethylene glycol-administered rats. Means which share the same letter(s) are not significantly different.

**Figure 24:**
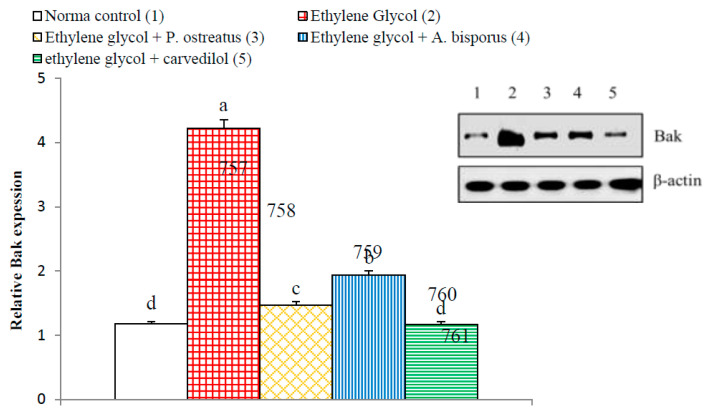
Effect of *P. ostreatus* and *A. bisporus* aqueous extracts and carvedilol on relative kidney Bak expression detected by Western blot in ethylene glycol-administered rats. Means which share the same letter(s) are not significantly different.

On the other hand, the apoptotic mediators Bax and Bak of Bcl-2 family showed a significant increase (*p* < 0.01; LSD) in ethylene glycol-induced hyperoxaluric rats recording increases of 241.61% and 259.79%, respectively, as compared with normal control. The treatment of hyperoxaluric rats with *P. ostreatus* and *A. bisporus* aqueous extracts and carvedilol successfully resulted in a significant decrease (*p* < 0.01; LSD) in the elevated NF-κB p65 and NF-κB p50 expression as compared with the ethylene glycol-induced hyperoxaluric control rats. Carvedilol was the most potent in decreasing the elevated Bax and Bak levels (Figure 23 and Figure 24, Figures S23 and S24).

## 4. Discussion

The two tested mushrooms *P. ostreatus* and *A. bisporus* were subjected to preliminary phytochemical screening and GC-MS analysis of their aqueous extracts. The phytochemical screening indicated the presence of glycosides, alkaloids, flavonoids, resins, tannins and unsaturated sterols. Most of these compounds were proven, by many publications, to have biological activities, e.g., antimicrobial, anti-inflammatory and antioxidant [64,65,66]. Moreover, many of the major compounds and chemical groups, detected in *P. ostreatus* aqueous extract by GC-MS, have been reported to have antioxidant and anti-inflammatory properties; these include (S)-(+)-isoleucinol [67], N-hydroxy-N-methyl-methanamine [66], phenylethanal [68,69] and 2-pyrrolidinone [70,71]. On the other hand, GC-MS of *A. bisporus* extract revealed the presence of multiple antioxidant and anti-inflammatory abundant constituents and chemical groups including N-methoxy-methanamine, [66], 2-pyrrolidinone [70,71] and fumaric acid, 2-heptyl octyl ester [72,73,74,75].

In the present study, the body weight significantly decreased in the ethylene glycol-supplemented control rats and the administration of aqueous extracts of *P. ostreatus* and *A. bisporus* as well as carvedilol to ethylene glycol-administered rats resulted in a remarkable increase in body weight. It is worth mentioning that the body weight loss is considered as a good reliable sensitive toxicity indicator [76,77,78]. Thus, the decrease in body weight, in the present study, by ethylene glycol may represent the first preliminary indicator of its toxicity. The prevention of decrease in body weight in ethylene glycol-administered rats by *P. ostreatus* and *A. bisporus* aqueous extracts and carvedilol may be due to their ability to antagonize the toxicity effects of ethylene glycol. The amendment of the biochemical and histological perturbations by the mushroom extracts and carvedilol, in the present study, may lead to improvement of general health and feeding ability. On the other hand, kidney weight and relative kidney weight in the ethylene glycol-administered rats exhibited a significant increase as compared with the normal control. That increase may be due to inflammation, fluid accumulation and crystal formation in the kidney as a result of ethylene glycol administration [28,79,80]. These results are in concordance with Wang et al. [81], Saeidi et al. [82] and Aggarwal et al. [83] publications, which demonstrated that the body weight was significantly reduced both in hyperoxaluric and lithogenic rats as compared with normal control rats while kidney weight and relative kidney weight showed a significant increase. In contrast, Noorafshan et al. [84] found that kidney weight and relative kidney weight decreased in ethylene glycol-administered control rats as compared with normal control rats. The treatment of ethylene glycol-induced hyperoxaluric rats with *P. ostreatus* and *A. bisporus* aqueous extracts and carvedilol, in the current study, did not significantly alter the kidney weight and relative kidney weight as compared with ethylene glycol-administered control.

In the present experiments, the administration of ethylene glycol led to a rise in serum creatinine, serum urea, serum uric acid and urine uric acid concentrations, as well as a decrease in urine creatinine, urine urea levels and ratios of urine creatinine/serum creatinine, urine urea/serum urea and Fex Urea. The decline in urine creatinine/serum creatinine, urine urea/serum urea and Fex urea ratios may be attributed to the diminution in creatinine, urea and uric acid clearance from blood to urine due to reduction in the glomerular filtration rate in addition to the damage in the renal tubular cells [85,86,87]. The results are in agreement with many investigators [13,88,89,90]. As to how these effects are caused, it was stated that drugs inducing nephrotoxicity are often associated with remarkable increases in acute tubular necrosis and blood urea [91]. In this regard, it has been shown that severe damage in proximal tubule cells may cause increases in serum creatinine and urea [87]. This damage appears to inhibit clearance of waste products, particularly nitrogenous substances such as blood creatinine, urea and uric acid, meaning that they accumulate in the blood [92,93,94]. The deteriorations in the biomarkers of kidney function and glomerular filtration rate as well clearance of waste by-products were concomitant with atrophy and congestion of glomeruli as well as necrosis and apoptosis of renal tubular cells as indicated in the present histological studies.

High levels of urine uric acid (evident in the present research, in the ethylene glycol control group) point to an increased risk of stone formation since urine uric acid is considered one of the most important promoters of crystallization [93]. This postulation aligns with the publication, which revealed that hyperuricosuria can be considered the main risk factor for the formation of urinary uric acid stones and may also play a role in CaOx lithiasis [95]. The predominance of uric acid crystals in CaOx stones and the observation that uric acid binding proteins are able to binding to CaOx to modify its crystallization reflect its essential role in the formation of stones [5,96,97].

The contribution of the present study is to show that seven weeks of treatment with aqueous extracts of *P. ostreatus* and *A. bisporus*, like treatment with carvedilol, reduced the elevation in serum creatinine, serum urea, serum uric acid and urine uric acid; *P. ostreatus* and *A. bisporus* seemed to be more potent than carvedilol. The treatments also produced an increase in the levels of urine creatinine, urine urea and ratios urine creatinine/serum creatinine and urine urea/serum urea as well as Fex Urea; *P. ostreatus* and *A. bisporus* appeared to be more effective. These changes may be attributed to the increased clearance of blood creatinine, urea and uric acid into urine. These treatment outcomes are consistent with other publications in different models of kidney injuries [86,98,99,100,101]. In our opinion, the improvements in serum and urine parameters and ratios related to kidney function as a result of treatments of hyperoxalouric rats with mushroom extracts and carvedilol may be secondary to their preventive effects against ethylene glycol-induced deteriorations in kidney histological architecture and integrity. The histological evidences, in the current study, support this elucidation since the treatments with mushroom extracts and carvedilol produced marked amelioration in kidney histological lesions produced by ethylene glycol supplementation.

Ethylene glycol administration, in the current study, caused a significant elevation in levels of calcium in the kidneys and in levels of oxalate in serum, urine and kidney. These findings parallel those of several researchers [13,102,103,104,105]. In addition, it was also elucidated by other investigators that the formation of stones in ethylene glycol-administered animals is caused by hyperoxaluria, which leads to elevation of both kidney excretion and retention of oxalate [106]. It is known from previous publications that an imbalance between the promoters of lithogenesis such as phosphate, oxalate, calcium, uric acid and low urine volume on the one hand and inhibitors including magnesium, citrate and macromolecules, on the other hand may represent a critical condition for urolithiasis [103,107]. Thus, the elevation of kidney oxalate and kidney calcium in association with the increase in uric acid, in the present study, activates the process of crystallization and precipitation of crystalline material as CaOx. In that manner, other past publications showed that the crystals of CaOx and high oxalate concentrations in nephrons damages epithelial cells, inducing nucleation of heterogeneous crystal and causing crystals aggregation [108,109]. In support to this evidence, the histological findings of the present study depicted the presence of oxalate crystals in the lumen of the renal tubules in association with inflammation and necrosis of renal tubules.

Treatment of ethylene glycol-administered rats with the tested mushroom extracts and carvedilol reduced the above risk factors. Thus, *P. ostreatus*, *A. bisporus* and carvedilol treatments led to reduced levels of serum and urine oxalate, which was associated with a decrease in the level of crystal components (calcium and oxalate) in kidney homogenate. These results are consistent with the present histological investigations, which depicted the absence of oxalate crystals in the renal tubules in ethylene glycol-induced urolithic rats treated with *P. ostreatus*, *A. bisporus* and carvedilol.

In association with the significant increase in urine uric acid and oxalate levels in the ethylene glycol-administered rats in the present study, the urine specific gravity significantly elevated in comparison with normal control. These results are in concordance with Kandhare et al. [110] and in discordance with Cruzan et al. [111]. The increase in urine specific gravity and urine uric acid and oxalate content in ethylene glycol-administered rats are important indices for the increased possibility for the formation of urinary calculi. On the other hand, the treatments of the ethylene glycol-administered rats with *P. ostreatus* and *A. bisporus* aqueous extracts and carvedilol produced a significant decrease in the specific gravity of urine towards the normal values. In our opinion, this decrease in specific gravity may be due to the resultant decrease in urine levels of uric acid and oxalate.

In the current study, administration of ethylene glycol, which leads to hyperoxaluria and nephrotoxicity, was evidenced by histopathological investigations, which showed the presence of marked atrophy in the glomerular tufts, protein and cellular casts, interstitial nephritis and many oxalate crystal precipitations in the lumina of the renal tubules. These results are in concordance with those of other authors who found tubular hypertrophy, interstitial inflammation, tubulointerstitial damage and extensive deposition of CaOx crystals in the renal tubules of different urolithiatic rat models [112,113,114,115,116]. In the present study, as a result of ethylene glycol administration, renal tubules exhibited cystic dilatation, focal necrosis associated with massive inflammatory cells infiltration, presence of *dysplasia* and *anaplasia* in the lining epithelium of some renal tubules, and infiltration of focal inflammatory cells and apoptotic cells in other renal tubules. These changes agree with the results obtained by Li et al. who observed severe dilatation of kidney tubules and massive inflammatory cell infiltration in ethylene glycol-administered rats [117]. Furthermore, Bashir and Gilani postulated that the dilatation of the renal tubules may be due to the obstruction in the distal renal tubular flow by large crystals [118]. In rats exposed to low levels of ethylene glycol, the renal damage (a proximal tubule cell necrosis) is directly related to the amount of accumulation of calcium oxalate in kidney tissue [111,119]. The necrotic damage is most likely due to the accumulation of calcium oxalate crystals, since, of the various ethylene glycol metabolites, only oxalate is cytotoxic to kidney cells in culture at relevant concentrations [120,121].

**Figure 25:**
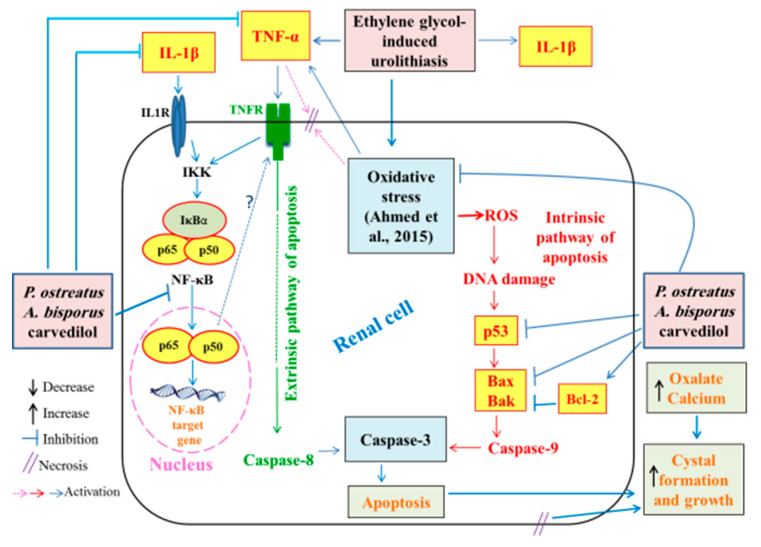
Schematic diagram showing the effects of *P. ostreatus* and *A. bisporus* extracts and carvedilol on NF-κB, p53 and Bcl-2 to suppress inflammation and apoptosis together with the effects on oxidative stress.

The inflammation observed in the histopathological observations in kidney of ethylene glycol-induced hyperoxaluric rats, in the present study, was confirmed by a significant increase in the levels of serum TNF-α and IL-1β as well as an increase in the kidney NF-κB, NF-κB p65 and NF-κB p50. The treatments of hyperoxaluric rats with *P. ostreatus* and *A. bisporus* aqueous extracts and carvedilol produced a significant decrease in these inflammatory mediators. Thus, the three treatments have potent anti-inflammatory potentials. It is relevant here to mention that TNF-α and IL-1β are T helper 1 (Th1) cytokines and are important stimuli for activation of NF-κB that is found in almost animal cell types including renal cells [122,123]. In an inactivated state, NF-κB in the cytosol is presented complexed with the inhibitory protein IκBα [124]. A variety of extracellular signals including TNF-α and IL-1β (canonical pathway) can activate the enzyme IκB kinase (IKK), which results in dissociation of NF-κB from IκBα and subsequent proteasomal degradation of IκBα [124,125]. This allows the release of the p65/p50 heterodimers and their translocation into the nucleus, where they can bind to their specific sequences of DNA and promote NF-κB target genes involved in inflammatory, immune and acute phase responses (Figure 25) [126,127,128]. Thus, based on the present results and previous literatures, it can be suggested that *P. ostreatus* and *A. bisporus* aqueous extracts and carvedilol may produce their anti-inflammatory effects by affecting the canonical pathway of NF-κB activation (Figure 25 and Figure S25).

Increased apoptosis evidenced, in the present study, by histopathological examination of kidney of ethylene glycol-induced hyperoxaluric rats, was supported by immunohistochemical and Western blot investigations, which revealed a remarkable increase in pro-apoptotic proteins p53, Bax and Bak as well as a decrease in the anti-apoptotic mediator, Bcl-2. The treatments of hyperoxaluric rats with *P. ostreatus* and *A. bisporus* aqueous extracts and carvedilol produced a significant decrease in such elevated pro-apoptotic proteins and increase in the lowered anti-apoptotic mediator Bcl-2 in the kidney. It is worth mentioning that p53, Bax and Bak as well as Bcl-2 are implicated in the intrinsic pathway of apoptosis that is activated by ROS produced by mitochondria (Figure 25 and Figure S25). The tumor suppressor p53 is a transcription factor that is activated upon DNA damage to induce target genes involved in either cell growth arrest or apoptosis [129,130,131]. The p53, Bax and Bak mediate intrinsic signaling pathway to activate caspase-9 that in turn activate caspase-3 leading to apoptosis (Figure 25 and Figure S25) [132]. This activation can be controlled by the Bcl-2, which has anti-apoptotic effects [130,131,132,133]. Otherwise, TNF-α activates the extrinsic pathway of apoptosis by its binding to TNF receptor (TNFR) and death receptor (Figure 25 and Figure S25). In addition, NF-κB was reported to have a role in the extrinsic pathway of apoptosis. NF-κB can oppose or promote apoptosis depending on the specific cell type and the type of inducer [134]. Although many publications reported the anti-apoptotic roles of NF-κB, many others revealed its pro-apoptotic and apoptotic actions in other circumstances [135,136,137,138,139]. Based on the findings of the present study and previous publications, it can be elucidated that *P. ostreatus* and *A. bisporus* aqueous extracts and carvedilol may produce their inhibitory effects on apoptosis by affecting both extrinsic and intrinsic pathways (Figure 25 and Figure S25).

Stone formation is related with the sedimentation of fragments in the peritubular field and in the medullary interstitium, and such sedimentation can create inflammation and aid the advancement of fibrosis, which follows tubular cell damage and is a determinant of kidney function [140]. In addition, ROS led to a robust inflammatory response in the kidneys of rats with hyperoxaluria and CaOx nephrolithiasis [5,28,141]. In this regard, it was suggested that renal tubular crystal deposition and apoptotic changes induced by hyperoxaluria play a role in the pathogenesis of urolithiasis [5,142]. In addition, it was speculated that renal tubular epithelial cell injury, apoptosis and inflammation are involved in melamine-related kidney stone formation [143]. Cell apoptosis and proliferation in the kidney cortex and medulla were enhanced in response to the glyoxylate-induced CaOx crystal formation and deposition [144]. In turn, apoptosis of renal tubular cells may promote stone formation via cellular demise and post-apoptotic necrosis, which could stimulate calcium oxalate crystal aggregation and growth as, indicated in Figure 25 and Figure S25 [5]. This postulation has been supported by in vitro study on Madin-Darby Canine Kidney (MDCK) cells which were exposed to oxalate ions [145]. Based on the previous findings and literature, it can be suggested that augmentation of inflammation and apoptosis in hyperoxaluric rats (Figure 25 and Figure S25) may have an important role in oxalate crystal formation and growth. Thus, the suppression of inflammation and apoptosis by treatment of hyperoxaluric rats with *P. ostreatus* and *A. bisporus* extracts and carvedilol may be implicated to reduce oxalate crystal formation and growth.

The treatment of ethylene glycol-administered rats with *P. ostreatus* and *A. bisporus* extracts and carvedilol led to decrease in renal expression of NF-κB, NF-κB p65, NF-κB p50, p53, Bax and Bak and increase in renal expression of Bcl-2, and it simultaneously prevented the ethylene glycol-induced histological deteriorations, inflammations and apoptosis in kidney. *P. ostreatus* and *A. bisporus* extracts were more effective on NF-κB and Bcl-2 than carvedilol while carvedilol was the most potent in decreasing the elevated kidney p53, NF-κB p50, Bax an Bak. These results are in accordance with those of many authors. Jedinak et al. concluded that *P. ostreatus* possesses anti-inflammatory activities and could be considered a dietary agent against inflammation [146]. Also, Amstad et al. elucidated that Bcl-2-overexpressing cells have an elevated expression of antioxidant enzymes and have higher levels of cellular GSH [147]. Besides, Volman et al. noticed that NF-κB transactivation in human intestinal Caco-2 cells was lowered by polysaccharide extract of *A. bisporus* [148]. Meanwhile, other investigators reported that the vasodilatory *β*-blocker carvedilol exhibits additional effects in ameliorating inflammation [149,150,151]. In another way, it was found that the pre-treatment of potassium dichromate-induced nephrotoxicity in rats with carvedilol caused a decrease in p53 and an increase in Bcl-2 expression [152].

In our previous study, we found that the treatment of ethylene glycol-administered rats with *P. ostreatus* and *A. bisporus* extracts and carvedilol produced potent inhibitory effects on kidney oxidative stress represented by decrease in lipid peroxidation and nitric oxide and potent enhancement in the kidney antioxidant defense system marked by increase in glutathione content and glutathione-S-transferase, glutathione peroxidase, superoxide dismutase and catalase activities [28]. This led us to suggest that the ameliorative effects on kidney function and histological integrity as well as anti-apoptotic and anti-inflammatory effects *P. ostreatus*, *A. bisporus* and carvedilol may be mediated, at least in part, via suppression of the oxidative stress and enhancement of antioxidant defense system (Figure 25 and Figure S25).

In conclusion, the present study demonstrated that *P. ostreatus* and *A. bisporus* aqueous extracts and carvedilol protected against ethylene glycol-induced rat kidney dysfunction, histological perturbances, oxalate crystal formation, inflammation and apoptosis. *P. ostreatus* and *A. bisporus* aqueous extracts were more potent than carvedilol in many instances. In spite of these potential effects, further clinical studies are required to estimate the safety and efficacy of *P. ostreatus* and *A. bisporus* extracts in human beings.

## Figures and Tables

**Table 1:** Preliminary phytochemical screening of *A. bisporus* and *P. ostreatus*.

	Mushroom	*P. ostreatus*	*A. bisporus*
Test			
**1—Carbohydrates and/or glycosides (Molish test)**		+ve	+ve
**2—Alkaloids and/or basic nitrogenous substances** (a) Dragendroff’s test (b) Mayer’s test (c) Wagner’s test			
		+ve	+ve
		+ve	+ve
		+ve	+ve
**3—Flavonoides** (a) Amyl alcohol test (b) Sodium hydroxide test			
		+ve	+ve
		−ve	−ve
**4—Resins test**		+ve	+ve
**5—Tannins** (a) Ferric chloride test (b) Hydrochloric acid test			
		−ve	−ve
		+ve	+ve
**6—Unsaturated sterols and/or triterpenes** (a) Libermann-Burchard test (b) Salkowiski’s test			
		+ve	+ve
		+ve	+ve

**Table 2:** Chemical composition of *P. ostreatus* aqueous extract as detected by GC-MS analysis.

Number	Retention Time (Minutes)	Compound or Group (From Central Library Search Report)	Area % (≥0.5%)
1	1.87	(S)-(+)-Isoleucinol	23.0
2	2.05	Diethylhydroxylamine	3.6
3	2.13	3-(dimethylamino)-propanenitrile	1.4
4	2.17	1-Methylcyclopropanemethanol	1.0
5	2.23	*N*-Butyl-formamide	3.5
6	2.54	2-(ethylthio) tetrahydro 2H-Pyran	4.0
7	2.72	*N*-Hydroxy-*N*-methyl methenamine	24.2
8	2.85	2-Ethyl-2-butenal	9.4
9	3.22	4-Methyl- 2,4,6-cycloheptatrien-1-one	6.4
10	3.29	2-Phenylethanal	4.2
11	3.47	2-Pyrrolidinone	4.4
12	3.79	3-Mercapto-1-propanol	4.0
13	4.30	*N,N,N′*-Trimethyl-1,3-propanediamine	3.4
14	4.54	2,5-Dihydro-3-methyl-furan	1.2
15	4.72	2,3-Dimethyl-pentanal	1.2
16	5.01	*N,N*-Diethyl-*N′, N′*-dimethyl-1,2-ethanediamine	3.6
17	6.26	4-Amino-1,2,5-Oxadiazole-3-carbonitrile	0.5
18	10.46	Thiomorpholine	0.5
19	11.11	2,3′-Dipyridyl	0.5

**Table 3:** Chemical composition of *A. bisporus aqueous extract* as detected by GC-MS analysis.

Number	Retention Time	Compound or Group (From Central Library Search Report)	Area % (≥0.5%)
1	2.69	N-Methoxymethanamine	30.6
2	3.19	Phenyl oxirane	5.8
3	3.27	2-Phenylethanal	2.6
4	3.44	2-Pyrrolidinone	7.9
5	3.78	2-Propenylidene cyclobutene	3.0
6	4.31	1-Methoxy-3-methyl- benzene	2.4
7	5.01	3-Pentanone	2.1
8	5.40	*N*-[4-Aminobutyl]aziridine	1.7
9	17.33	*N*-Methoxy-methanamine	4.2
10	25.88	2,4,6,7,8,8a-Hexahydro-3,8-dimethyl-4-(1-methylethylidene)-(8S-cis)-5(1H)-azulenone	3.2
11	30.45	6-Ethyl-2,3-dihydro-2,7-dimethyl-5-oxo-5H-oxazolo[3,2-a]pyridine-8-carbonitrile	13.7
12	31.41	Oxime, (5. alpha.) androstan-3-one	6.5
13	31.92	6-Amino-2-Phenazinol	2.1
14	32.99	Fumaric acid, 2-heptyl octyl ester	13.1

## Supplementary Materials

The following are available online at https://www.mdpi.com/2218-273X/10/9/1317/s1, Figure S1: *P. ostreatus* (A) and *A. bisporus* (B); Figure S2: GC-MS analysis of *P. ostreatus* aqueous extract; Figure S3: GC-MS analysis of *A. bisporus* aqueous extract; Figure S4: Effect of *P. ostreatus* and *A. bisporus* aqueous extracts and carvedilol on body weight, kidney weight and relative kidney weight of ethylene glycol-administered rats.; Figure S5: Effect of *P. ostreatus* and *A. bisporus* aqueous extracts and carvedilol on levels of serum parameters related to kidney function of ethylene glycol-administered rats; Figure S6: Effect of *P. ostreatus* and *A. bisporus* aqueous extracts and carvedilol on different urine parameters related to renal efficiency of ethylene glycol-administered rats; Figure S7: Effect of *P. ostreatus* and *A. bisporus* aqueous extracts and carvedilol on various ratios related to kidney function of ethylene glycol-administered rats; Figure S8: Effect of *P. ostreatus* and *A. bisporus* aqueous extracts and carvedilol on serum, kidney and urine oxalate levels in ethylene glycol-administered rats; Figure S9: Effect of *P. ostreatus* and *A. bisporus* aqueous extracts and carvedilol on urine specific gravity in ethylene glycol-administered rats; Figure S10: Effect of *P. ostreatus* and *A. bisporus* aqueous extracts and carvedilol on kidney calcium content in ethylene glycol-administered rats; Figure S11: Effect of *P. ostreatus* and *A. bisporus* aqueous extracts and carvedilol on serum TNF-α and IL-1β levels in ethylene glycol-administered rats; Figure S12: Photomicrographs of hematoxylin and eosin stained sections of kidney of a normal control rat; Figure S13: Photomicrographs of hematoxylin and eosin stained sections of kidney of ethylene glycol control rat; Figure S14: Photomicrographs of hematoxylin and eosin stained sections of kidney of ethylene glycol-administered rat treated with *P. ostreatus* aqueous extract; Figure S15: Photomicrographs of hematoxylin and eosin stained sections of kidney of ethylene glycol-administered rat treated with *A. bisporus* aqueous extract; Figure S16: Photomicrographs of hematoxylin and eosin stained sections of kidney of ethylene glycol-administered rat treated with carvedilol; Figure S17: Photomicrographs (a–e) of immunohistochemically stained kidney sections for detection of NF-κB in different groups; Figure S18: Photomicrographs (a–e) of immunohistochemically stained kidney sections for detection of p53; Figure S19: Photomicrographs of immunohistochemically stained kidney sections for detection of Bcl-2; Figure S20: Effect of *P. ostreatus* and *A. bisporus* aqueous extracts and carvedilol on percent area of yellowish brown color of NF-κB, p53 and Bcl-2 expressions; Figure S21: Effect of *P. ostreatus* and *A. bisporus* aqueous extracts and carvedilol on relative kidney NF-κB p65 expression detected by Western blot in ethylene glycol-administered rats; Figure S22: Effect of *P. ostreatus* and *A. bisporus* aqueous extracts and carvedilol on relative kidney NF-κB p50 expression detected by Western blot in ethylene glycol-administered rats; Figure S23: Effect of *P. ostreatus* and *A. bisporus* aqueous extracts and carvedilol on relative kidney Bax expression detected by Western blot in ethylene glycol-administered rats; Figure S24: Effect of *P. ostreatus* and *A. bisporus* aqueous extracts and carvedilol on relative kidney Bak expression detected by Western blot in ethylene glycol-administered rats; Figure S25: Schematic diagram showing the effects of *P. ostreatus* and *A. bisporus* extracts and carvedilol on NF-κB, p53 and Bcl-2 to suppress inflammation and apoptosis together with the effects on oxidative stress.
