# Supplementary figures and images for: Nephroprotective Effect of Pleurotus ostreatus and Agaricus bisporus Extracts and Carvedilol on Ethylene Glycol-Induced Urolithiasis: Roles of NF-κB, p53, Bcl-2, Bax and Bak
Source: Biomolecules. 2020 Sep 14;10(9):1317. doi: 10.3390/biom10091317 (PMC7565610; doi:10.3390/biom10091317)

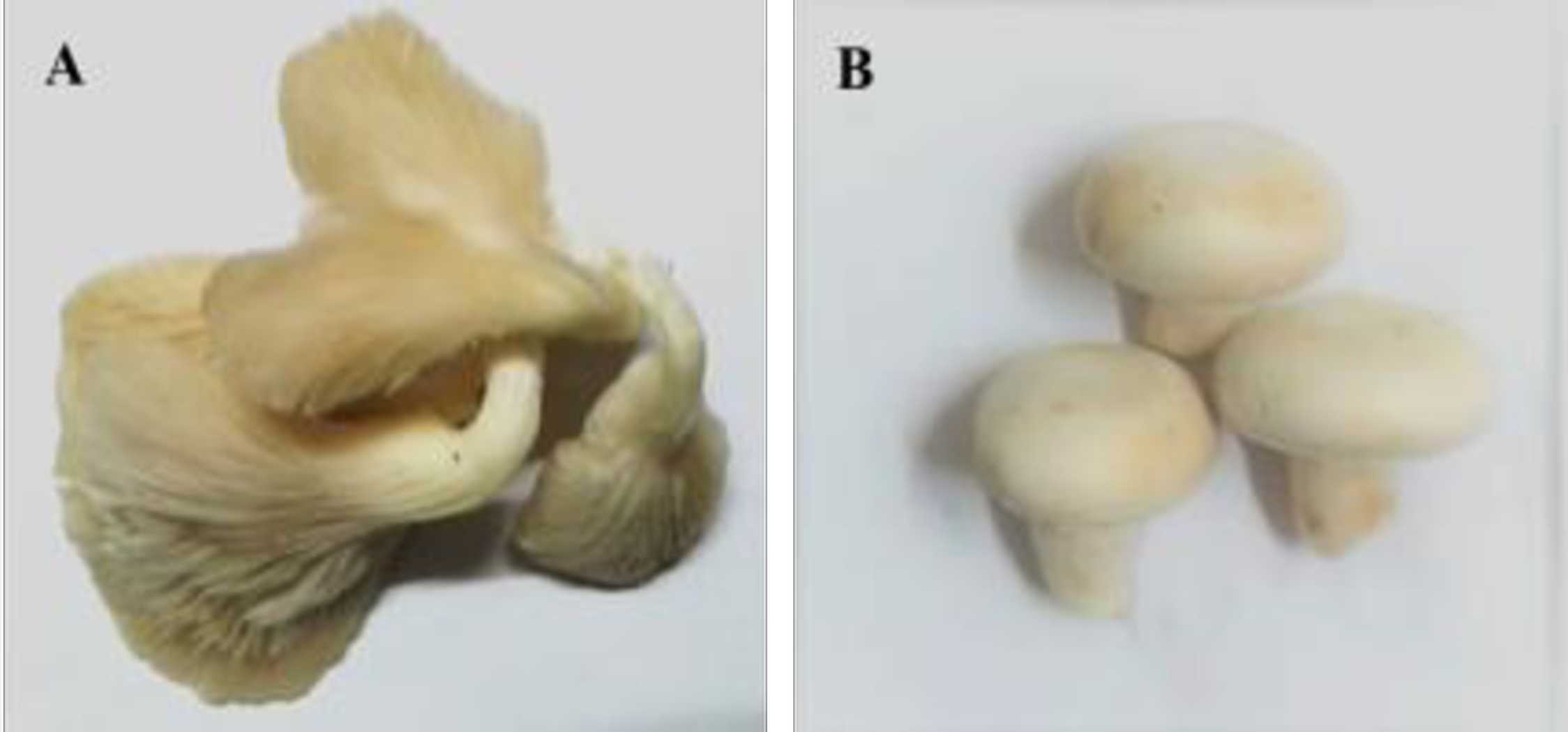

Supplement: Supplementary file 1 [file biomolecules-10-01317-s001.zip › Figures - Supplementary files/Figure S1.jpg]

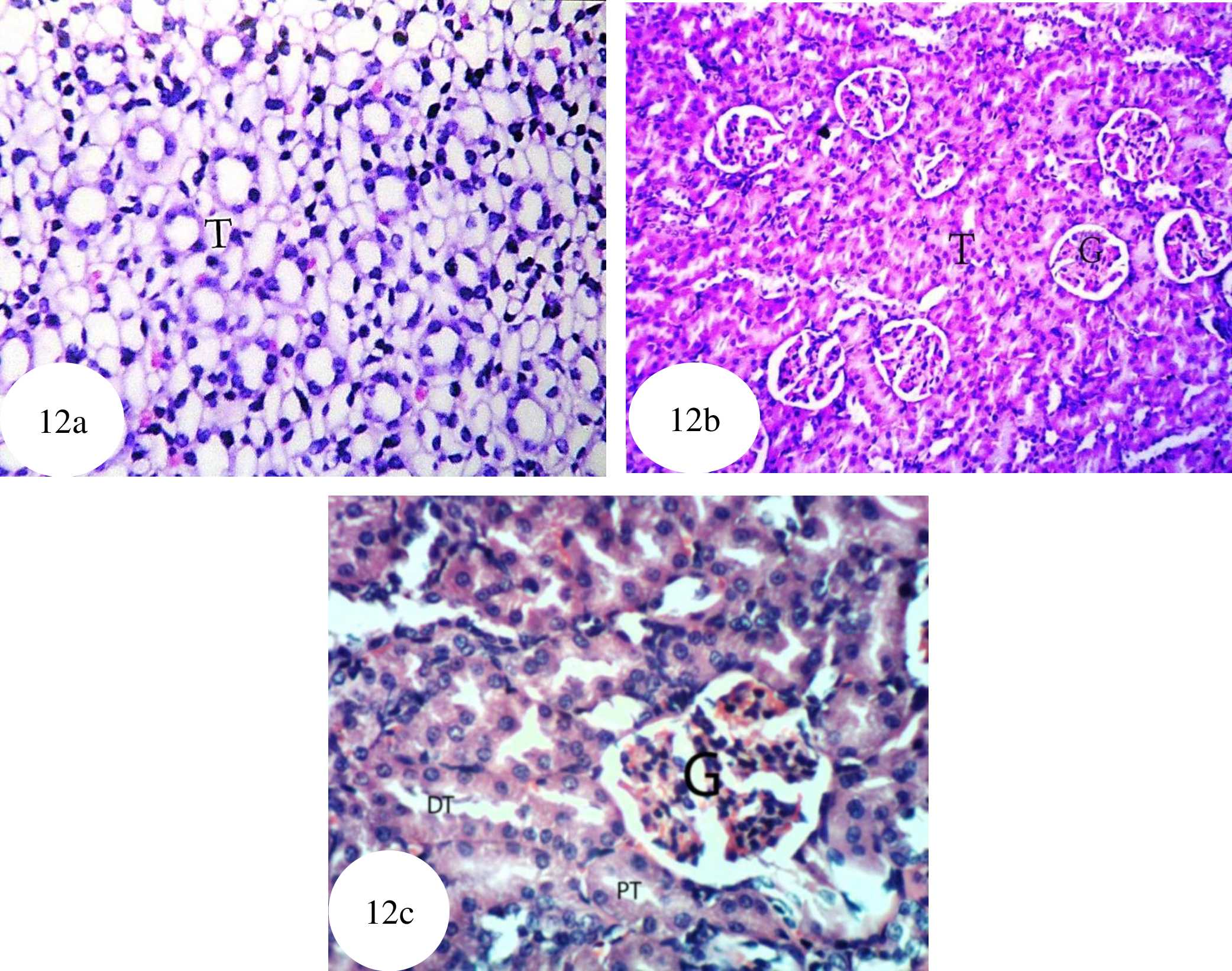

Supplement: Supplementary file 1 [file biomolecules-10-01317-s001.zip › Figures - Supplementary files/Figure S12.jpg]

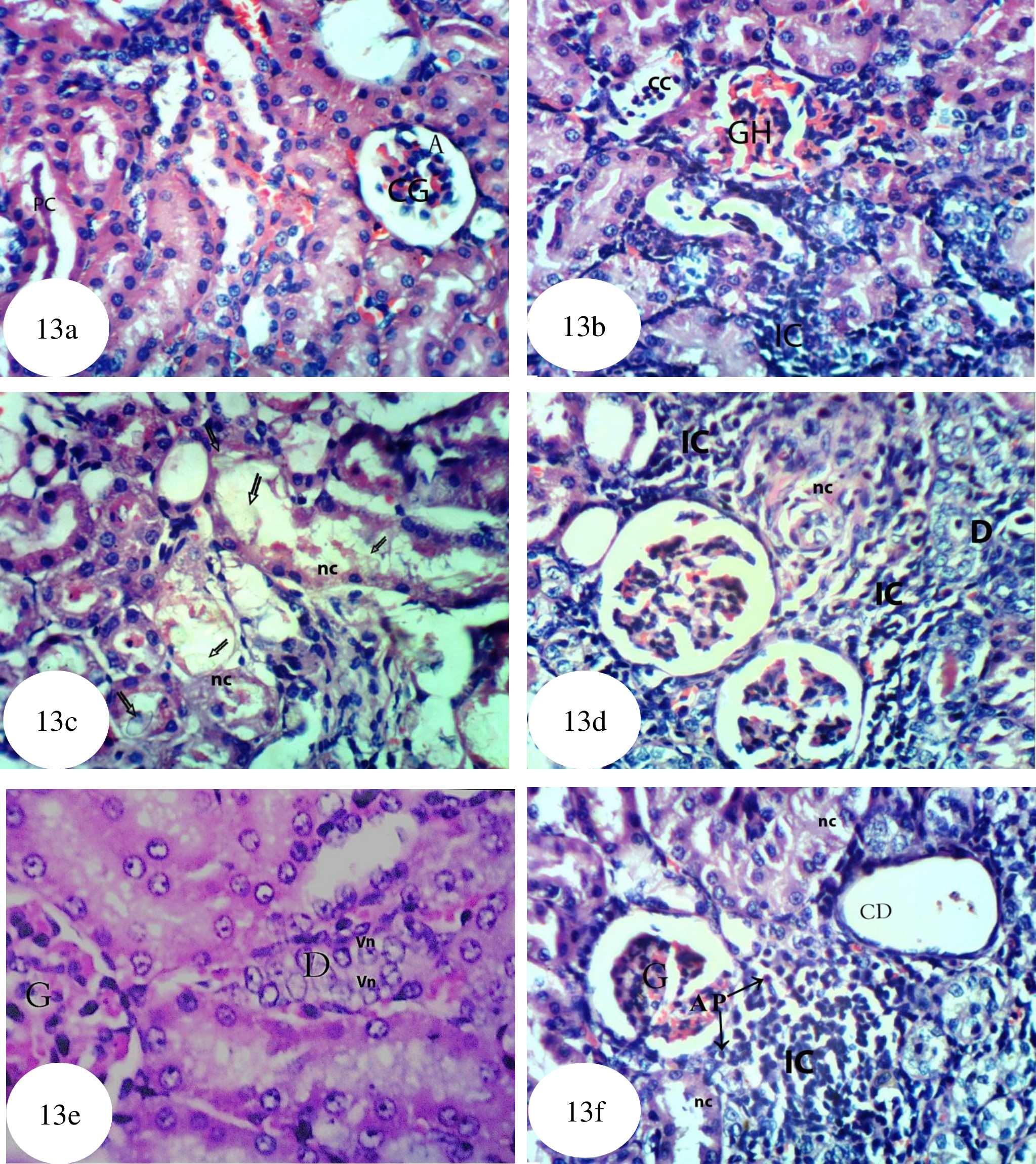

Supplement: Supplementary file 1 [file biomolecules-10-01317-s001.zip › Figures - Supplementary files/Figure S13.jpg]

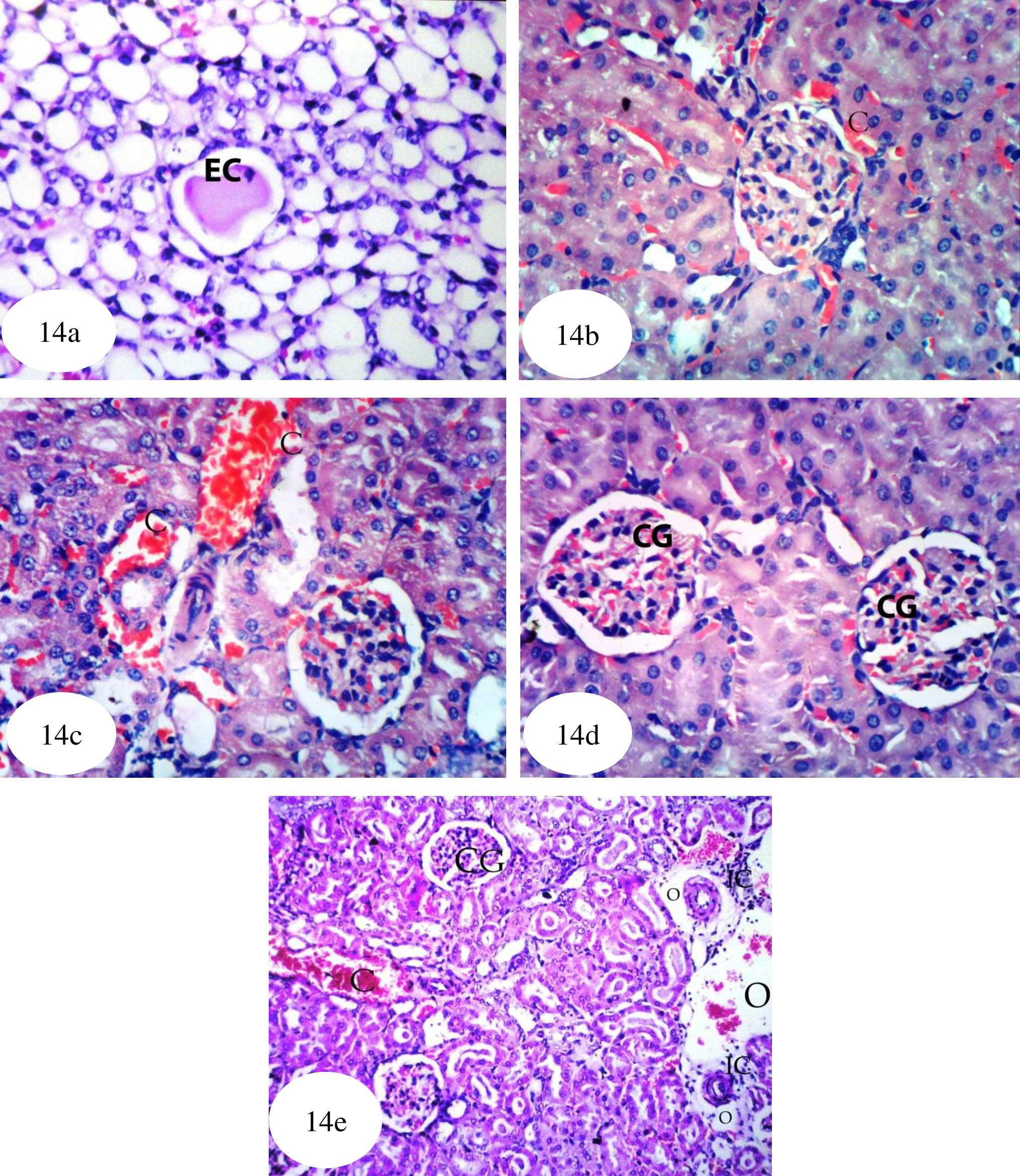

Supplement: Supplementary file 1 [file biomolecules-10-01317-s001.zip › Figures - Supplementary files/Figure S14.jpg]

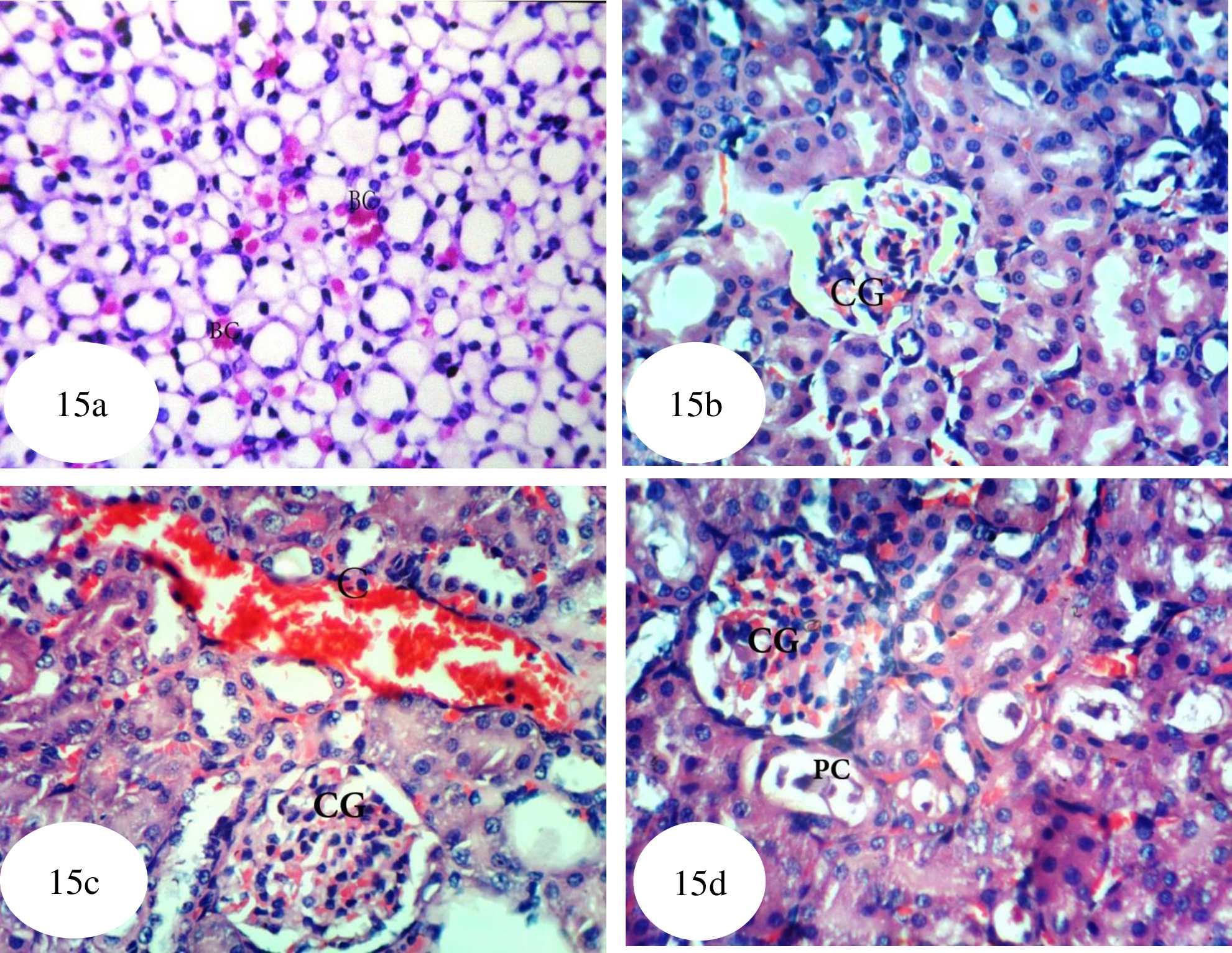

Supplement: Supplementary file 1 [file biomolecules-10-01317-s001.zip › Figures - Supplementary files/Figure S15.jpg]

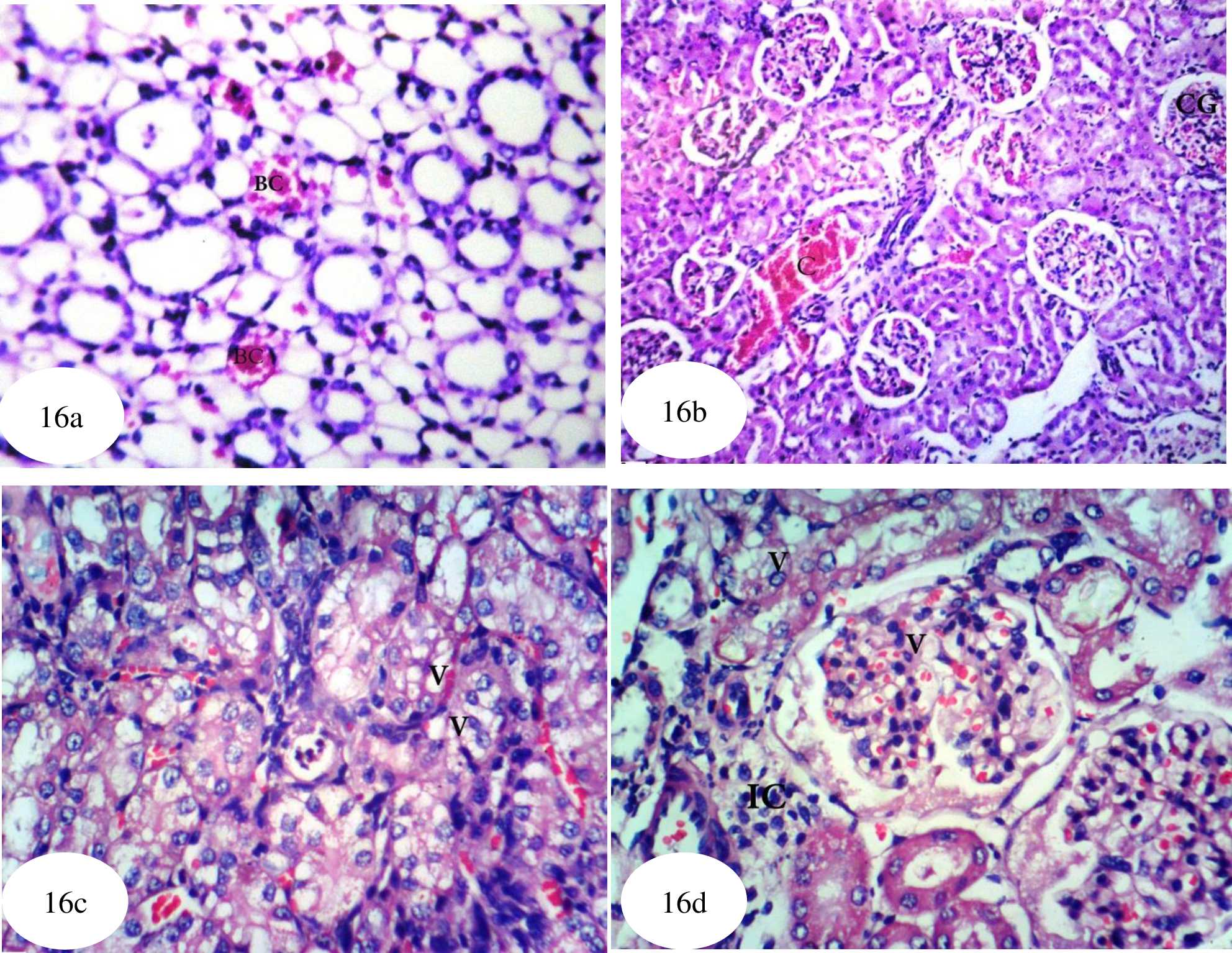

Supplement: Supplementary file 1 [file biomolecules-10-01317-s001.zip › Figures - Supplementary files/Figure S16.jpg]

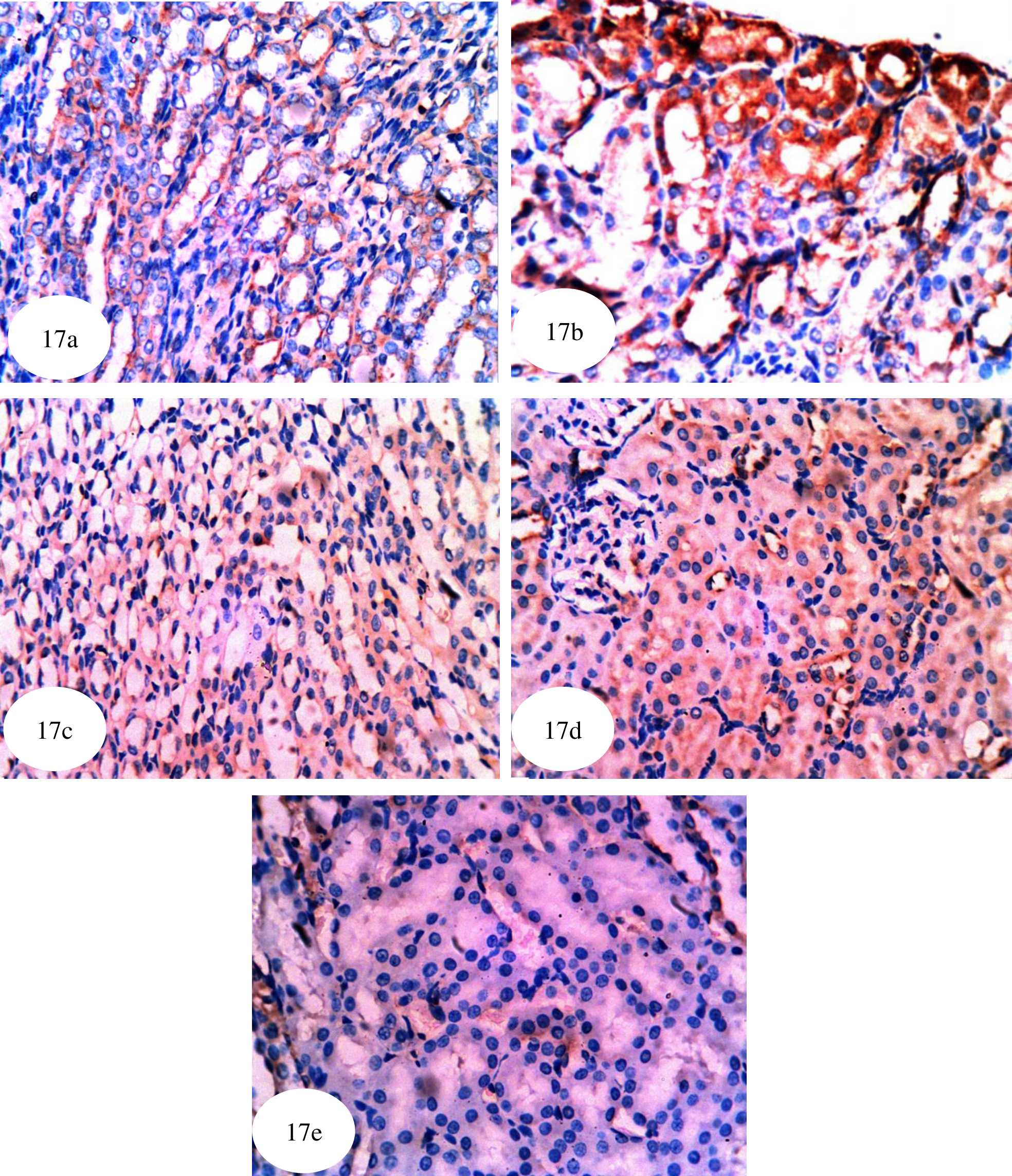

Supplement: Supplementary file 1 [file biomolecules-10-01317-s001.zip › Figures - Supplementary files/Figure S17.jpg]

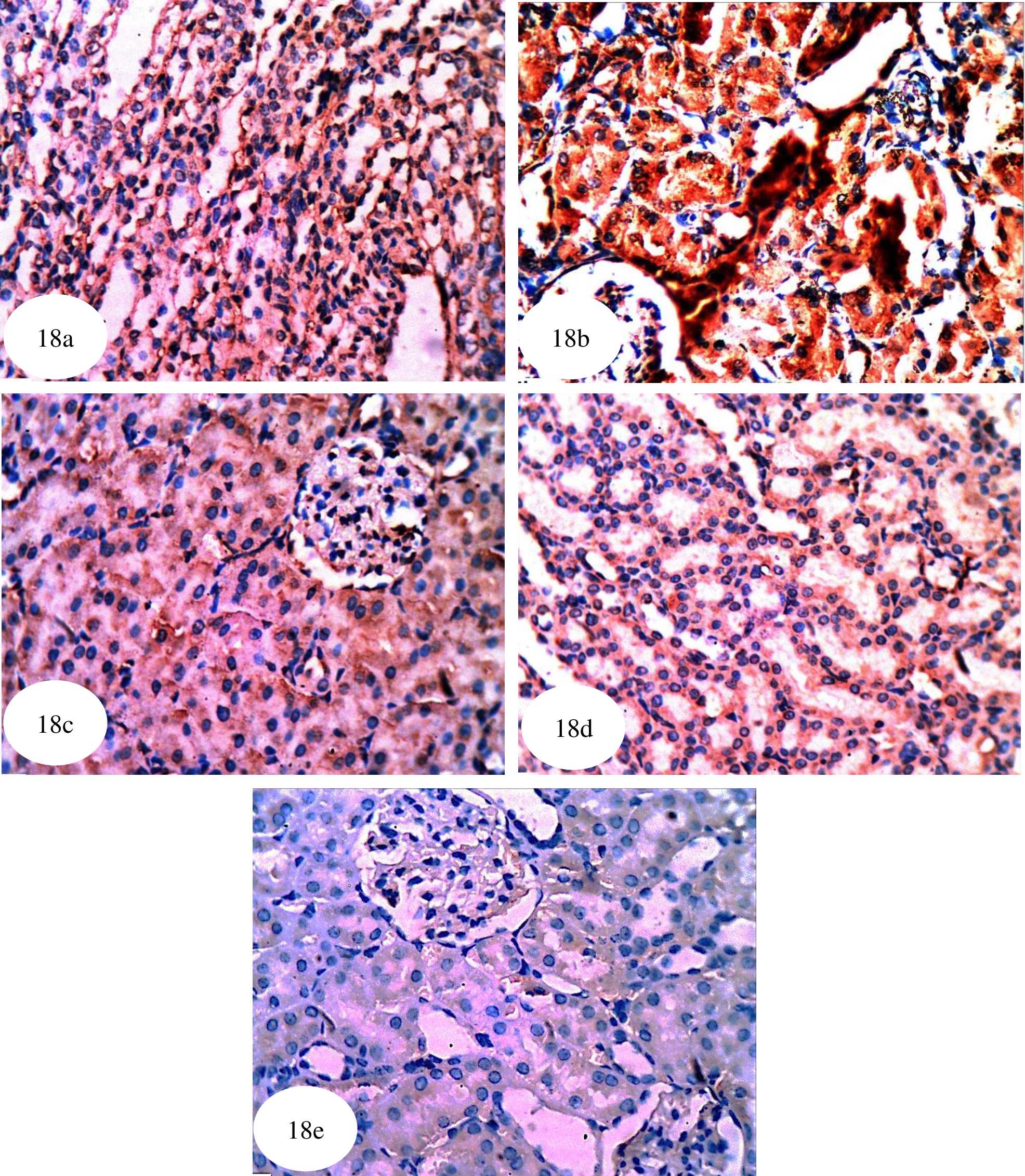

Supplement: Supplementary file 1 [file biomolecules-10-01317-s001.zip › Figures - Supplementary files/Figure S18.jpg]

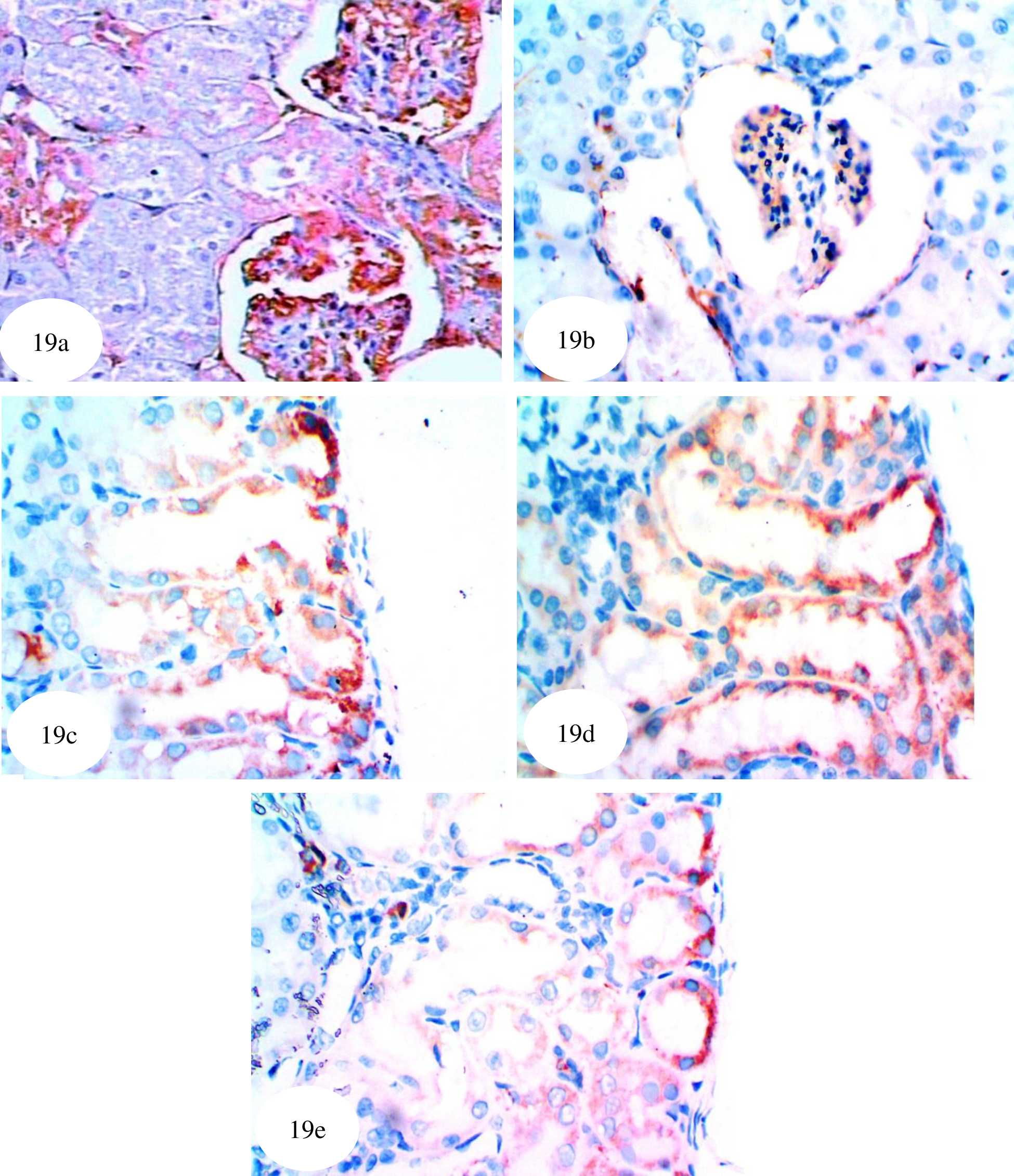

Supplement: Supplementary file 1 [file biomolecules-10-01317-s001.zip › Figures - Supplementary files/Figure S19.jpg]

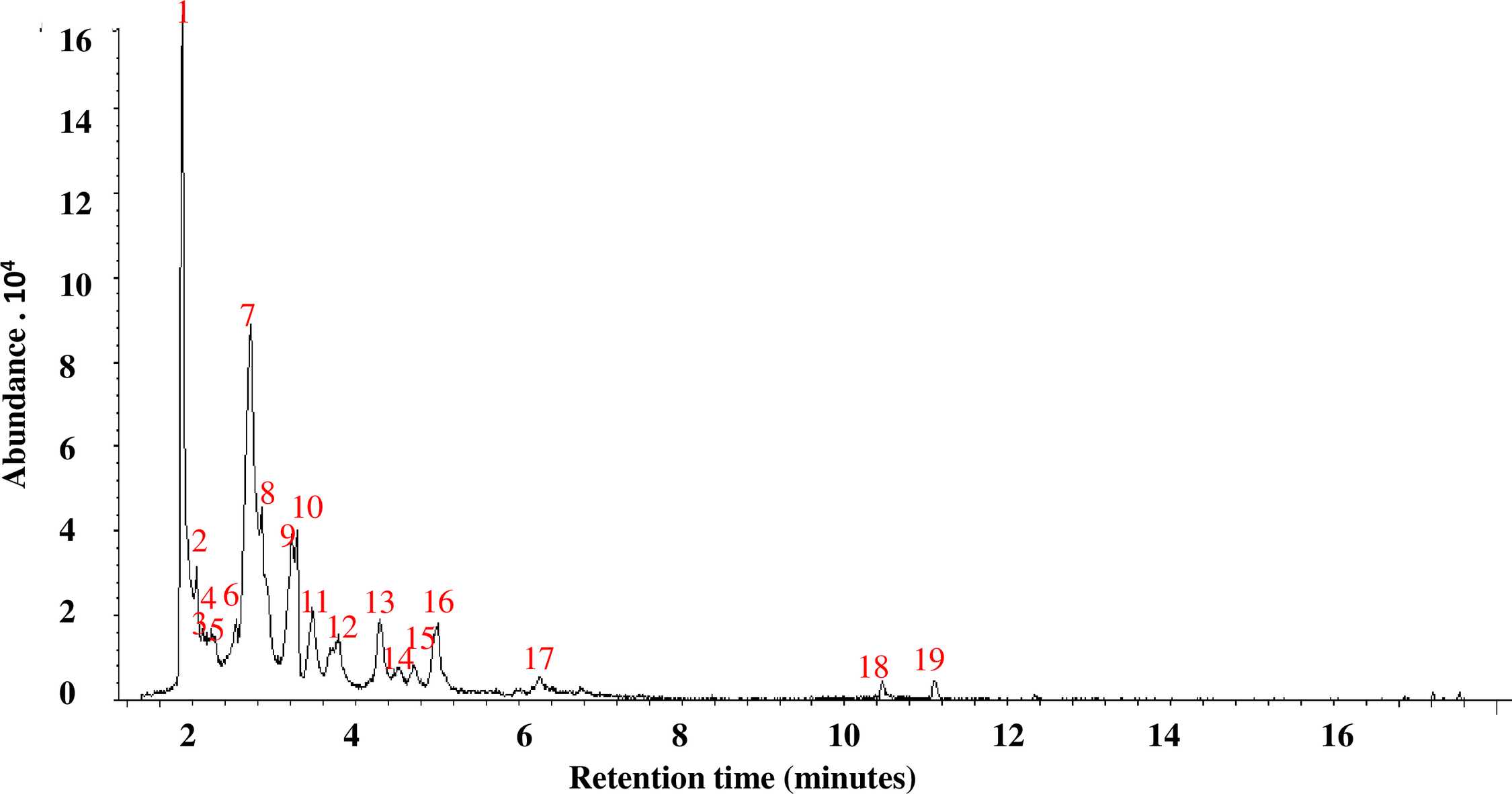

Supplement: Supplementary file 1 [file biomolecules-10-01317-s001.zip › Figures - Supplementary files/Figure S2.jpg]

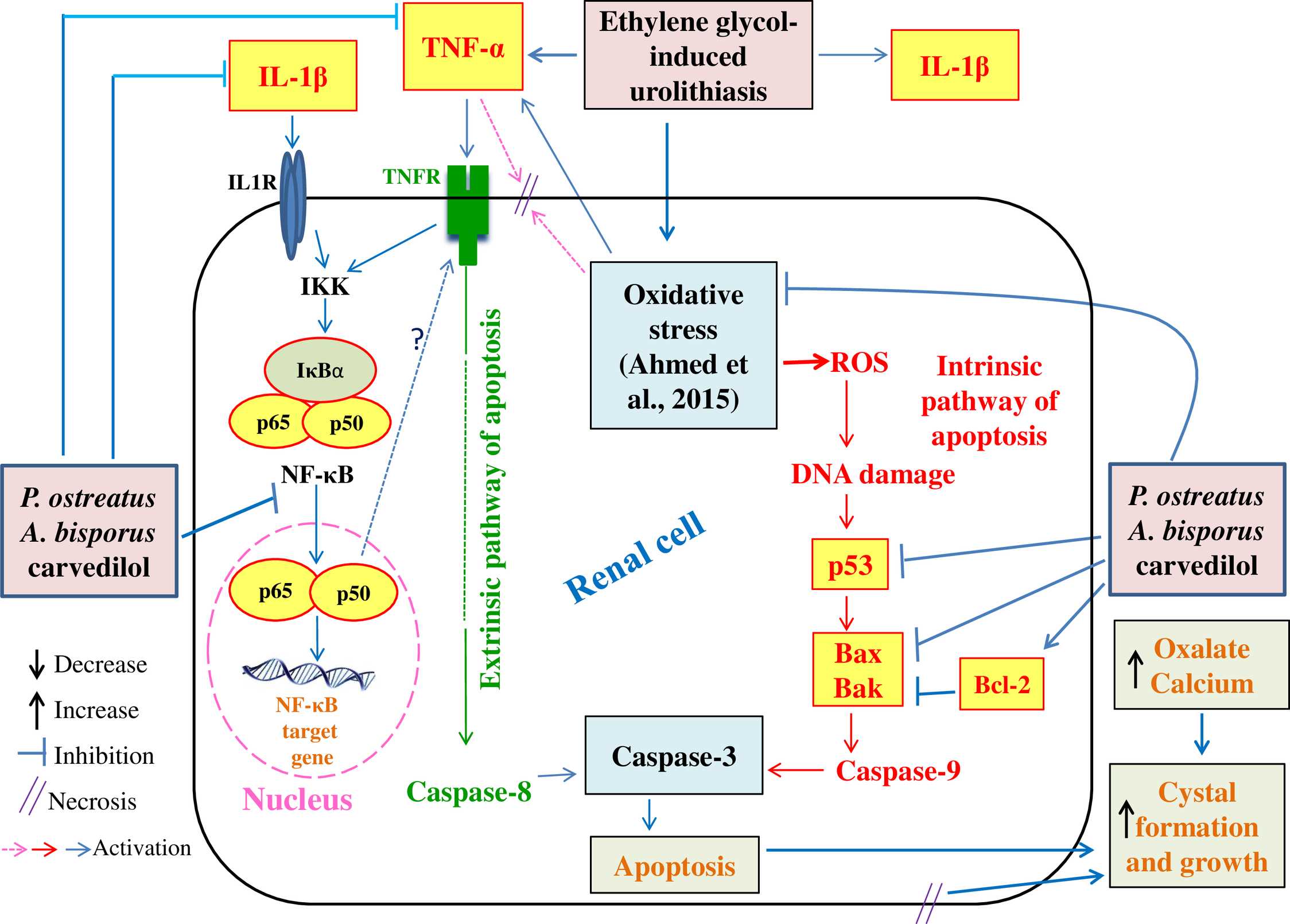

Supplement: Supplementary file 1 [file biomolecules-10-01317-s001.zip › Figures - Supplementary files/Figure S25.jpg]

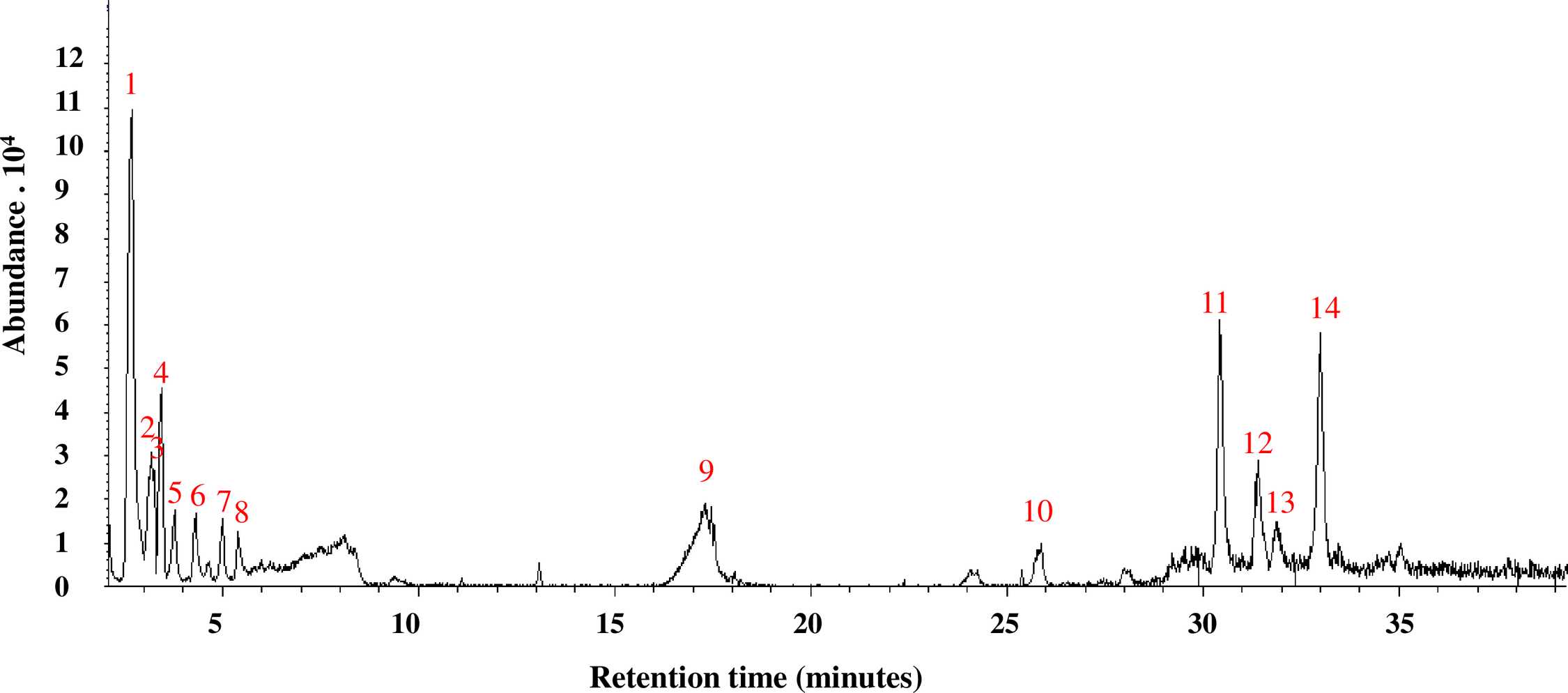

Supplement: Supplementary file 1 [file biomolecules-10-01317-s001.zip › Figures - Supplementary files/Figure S3.jpg]
